# Novel circular single-stranded DNA viruses identified in marine invertebrates reveal high sequence diversity and consistent predicted intrinsic disorder patterns within putative structural proteins

**DOI:** 10.3389/fmicb.2015.00696

**Published:** 2015-07-10

**Authors:** Karyna Rosario, Ryan O. Schenck, Rachel C. Harbeitner, Stephanie N. Lawler, Mya Breitbart

**Affiliations:** College of Marine Science, University of South FloridaSt. Petersburg, FL, USA

**Keywords:** single-stranded DNA virus, CRESS-DNA virus, circular DNA virus, intrinsically disordered proteins (IDPs), intrinsically disordered regions (IDRs), marine invertebrate, crustaceans

## Abstract

Viral metagenomics has recently revealed the ubiquitous and diverse nature of single-stranded DNA (ssDNA) viruses that encode a conserved replication initiator protein (Rep) in the marine environment. Although eukaryotic circular Rep-encoding ssDNA (CRESS-DNA) viruses were originally thought to only infect plants and vertebrates, recent studies have identified these viruses in a number of invertebrates. To further explore CRESS-DNA viruses in the marine environment, this study surveyed CRESS-DNA viruses in various marine invertebrate species. A total of 27 novel CRESS-DNA genomes, with Reps that share less than 60.1% identity with previously reported viruses, were recovered from 21 invertebrate species, mainly crustaceans. Phylogenetic analysis based on the Rep revealed a novel clade of CRESS-DNA viruses that included approximately one third of the marine invertebrate associated viruses identified here and whose members may represent a novel family. Investigation of putative capsid proteins (Cap) encoded within the eukaryotic CRESS-DNA viral genomes from this study and those in GenBank demonstrated conserved patterns of predicted intrinsically disordered regions (IDRs), which can be used to complement similarity-based searches to identify divergent structural proteins within novel genomes. Overall, this study expands our knowledge of CRESS-DNA viruses associated with invertebrates and explores a new tool to evaluate divergent structural proteins encoded by these viruses.

## Introduction

Viral metagenomics, or shotgun sequencing of total nucleic acids from purified virus particles, enables examination of viral communities without prior knowledge of the viruses present, thus resulting in an unprecedented view of viral diversity (Breitbart et al., 2002; Edwards and Rohwer, 2005; Angly et al., 2006). This technique has uncovered many novel viral types and extended the environmental distribution of known viral groups (Delwart, 2007; Rosario and Breitbart, 2011). In particular, the incorporation of rolling circle amplification (RCA) into viral metagenomic studies has unearthed a high diversity and wide distribution of eukaryotic viruses with circular, single-stranded DNA (ssDNA) genomes that encode a conserved replication initiator protein (Rep; Delwart and Li, 2012; Rosario et al., 2012a). Before the metagenomics era, eukaryotic circular Rep-encoding ssDNA (CRESS-DNA) viruses were only known in agricultural and medical fields since they are known plant (*Geminiviridae* and *Nanoviridae*) and vertebrate (*Circoviridae*) pathogens. However, over the past decade metagenomic approaches have revealed the ubiquitous nature of eukaryotic CRESS-DNA viruses, with reports from various environments, including deep-sea vents (Yoshida et al., 2013), Antarctic lakes and ponds (López-Bueno et al., 2009; Zawar-Reza et al., 2014), wastewater (Rosario et al., 2009b; Roux et al., 2013; Kraberger et al., 2015; Phan et al., 2015), freshwater lakes (Roux et al., 2012, 2013), oceans (Rosario et al., 2009a; Labonte and Suttle, 2013; Roux et al., 2013), hot springs (Diemer and Stedman, 2012), the near-surface atmosphere (Whon et al., 2012; Roux et al., 2013), and soils (Kim et al., 2008; Reavy et al., 2015). Novel CRESS-DNA viruses have also been discovered from fecal samples of a variety of vertebrates (Blinkova et al., 2010; Li et al., 2010a,b; Phan et al., 2011; Ge et al., 2012; Ng et al., 2012; Sachsenroder et al., 2012; van den Brand et al., 2012; Cheung et al., 2013, 2014; Sikorski et al., 2013a; Garigliany et al., 2014; Lian et al., 2014; Smits et al., 2014; Zhang et al., 2014; Sasaki et al., 2015). Notably, CRESS-DNA viruses similar to circoviruses, which were previously thought to only infect vertebrates, have now been identified in a myriad of invertebrates, including insects (Ng et al., 2011; Rosario et al., 2011, 2012b; Dayaram et al., 2013; Padilla-Rodriguez et al., 2013; Pham et al., 2013a,b; Garigliany et al., 2015), crustaceans (Dunlap et al., 2013; Hewson et al., 2013a,b; Ng et al., 2013; Pham et al., 2014), cnidarians (Soffer et al., 2014), and gastropods (Dayaram et al., 2015a), suggesting that CRESS-DNA viruses may be prevalent amongst unexplored taxa.

Well-studied viruses from the *Circoviridae*, *Nanoviridae*, and *Geminiviridae* families demonstrate the rapid evolutionary potential of CRESS-DNA viruses due to high nucleotide substitution rates (Duffy et al., 2008; Duffy and Holmes, 2009) as well as mechanistic predispositions to recombination (Lefeuvre et al., 2009; Martin et al., 2011). These characteristics, combined with the high level of recently reported diversity, highlight the need to continually revisit taxonomic classification of this viral group to add new species, genera and/or families. However, this task is complicated by the fact that many of the CRESS-DNA virus genomes exhibit novel genome architectures, only share similarities to the highly conserved Rep of known viruses, and have similarities to viruses belonging to multiple different taxonomic groups (Rosario et al., 2012a; Roux et al., 2013). In addition, the definitive hosts for many of these CRESS-DNA viruses remain unknown, hindering their classification according to traditional standards.

CRESS-DNA viruses are characterized by small genomes (∼1.7–3 kb) that contain 2–6 protein-encoding genes. The smallest monopartite CRESS-DNA viruses, members of the *Circoviridae* family, exhibit only two major open reading frames (ORFs), which encode a Rep and a capsid protein (Cap). Many of the novel eukaryotic CRESS-DNA viral genomes obtained from environmental samples or individual organisms through either metagenomic sequencing or degenerate PCR (herein referred to as “metagenomic CRESS-DNA viruses”) exhibit similarities to circoviruses and have been referred to as ‘circo-like’ viruses. Although many of the metagenomic circo-like virus genomes are highly divergent, these surveys have uncovered a novel CRESS-DNA viral group, the proposed Cyclovirus genus (Li et al., 2010a). Cycloviruses, which form a sister group to the *Circovirus* genus within the family *Circoviridae*, have been identified from both vertebrates (Li et al., 2010a; Smits et al., 2013; Tan Le et al., 2013; Garigliany et al., 2014; Zhang et al., 2014) and invertebrates (Rosario et al., 2011, 2012b; Dayaram et al., 2013, 2014, 2015b; Padilla-Rodriguez et al., 2013).

Similarities to circoviruses are mainly based on the Rep whereas the second major ORF in novel circo-like metagenomic CRESS-DNA viruses generally does not have any significant matches in the database but is assumed to encode for a structural protein based on the genomic architecture of known circoviruses. In lieu of significant matches to known structural proteins in the GenBank database, it is important to investigate putative novel Caps in CRESS-DNA viruses to provide evidence regarding their structural function. A potential avenue to identify conserved patterns in highly divergent structural proteins, such as those observed in novel metagenomic CRESS-DNA viruses, is to investigate the presence of predicted intrinsically disordered regions (IDRs). IDRs are regions within a protein that lack a rigid or fixed (i.e., ordered) structure, allowing a protein to exist in different states depending on the substrate with which it is interacting (Dunker et al., 2001; Brown et al., 2011). Research examining IDRs within viral proteomes has revealed that smaller viral genomes, such as those of CRESS-DNA viruses, contain a higher proportion of predicted disordered residues than larger viruses (Xue et al., 2012, 2014; Pushker et al., 2013). Therefore it has been suggested that small viruses may exploit IDRs to encode multifunctional proteins (Xue et al., 2012, 2014; Pushker et al., 2013). Since structural proteins in several viral families commonly contain IDRs (Chen et al., 2006; Goh et al., 2008a,b; Chang et al., 2009; Jensen et al., 2011), the presence of similar patterns of predicted disorder amongst unidentified CRESS-DNA proteins may provide one line of evidence for these proteins representing putative Caps.

To contribute to efforts exploring the diversity of CRESS-DNA viruses in invertebrates, this study investigated various marine invertebrate species for the presence of these viruses. A total of 27 novel CRESS-DNA genomes were recovered from 21 invertebrate species, expanding the known diversity of CRESS-DNA viruses associated with marine organisms and providing the first evidence of viruses associated with some under-sampled taxa. The well-conserved Rep of CRESS-DNA viruses was used to explore the relationships between these novel viruses and previously reported eukaryotic CRESS-DNA viruses in GenBank, including metagenomic CRESS-DNA viruses. In addition, the non-Rep-encoding ORFs (i.e., putative Caps) within these genomes were investigated for IDRs. Disorder prediction methods suggest that CRESS-DNA viral Caps exhibit conserved patterns of predicted disorder, which can be used to complement similarity-based searches to identify structural proteins within novel CRESS-DNA viral genomes.

## Materials and Methods

### Sample Processing and Genome Discovery

CRESS-DNA viruses were investigated in a variety of marine invertebrate species that were collected as samples of opportunity (**Table 1** and Supplementary Table S1). Specimens were identified with the highest degree of taxonomic resolution possible based on morphology. Whole organisms or tissue sections were serially rinsed three times using sterile SM Buffer [0.1 M NaCl, 50 mM Tris-HCl (pH 7.5), 10 mM MgSO_4_]. Viral particles were partially purified from each specimen prior to DNA extraction. For this purpose, samples were homogenized in one of two ways depending on the size of the specimen. Smaller organisms or dissected tissues that could be placed in a 1.5 ml microcentrifuge tube were homogenized in 1 ml of sterile SM Buffer through bead-beating using 1.0 mm sterile glass beads in a bead beater (Biospec Products). Homogenates were then centrifuged at 6000 × *g* for 6 min. Larger organisms or tissues of dissected organisms, such as muscle or gonads, were placed in a gentleMACS^TM^ M tube (Miltenyl Biotec) containing 3 ml of sterile SM buffer. Samples were then homogenized using a gentleMACS dissociator (Miltenyl Biotec) followed by centrifugation at 6000 × *g* for 9 min. The supernatant from both homogenization methods was filtered through a 0.45 μm Sterivex filter (Millipore) and nucleic acids were extracted from 200 μl of filtrate using the QIAmp MinElute Virus Spin Kit (Qiagen).

**Table 1:** CRESS-DNA genomes identified in this study, the organism they were obtained from, and genome details (acronym, genome length, nonanucleotide motif, genome type, and ORFs identified).

Genome^1^	Organism	Tissue type	Genome (bp)	Genomic architecture	Nonanucleotide^2^	Cap^3^	Rep
*P. diogenes* Giant Hermit Crab aCV(I0004A)	*Petrochirus diogenes*	Abdomen	1815	Type V	TAGTATTAC	X^∗^	X
*Palaemonete* sp. Common Grass Shrimp aCV (I0006H)	*Palaemonete* sp.	Hepatopancreas	2257	Type II	TAGTATTAC	X^∗^	X
*Aiptasia* sp. Sea Anemone aCV (I0007C2)	*Aiptasia* sp.	Whole organism	1901	Type I	CATTATTAC	X	X
*Aiptasia* sp. Sea Anemone aCV (I0007C3)	*Aiptasia* sp.	Whole organism	1942	Type I	CATTATTAC	X	X
*L. variegatus* Variable Sea Urchin aCV (I0021)	*Lytechinus variegatus*	Gonads	2167	Type III	GACTATTAC^∗^	X^∗^	X
*Didemnum* sp. Sea Squirt aCV (I0026A4)	*Didemnum* sp.	Whole organism	2061	Type IV	CAGTATTAC	X	X
*Didemnum* sp. Sea Squirt aCV (I0026A7)	*Didemnum* sp.	Whole organism	2143	Type I	CAGTATTAC	X^∗^	X
*Littorina* sp. Snail aCV (I0041)	*Littorina* sp.	Whole organism	2237	Type II	CAGTATTAC	X	X
*C. ornatus* Ornate Blue Crab aCV (I0054)	*Callinectes ornatus*	Gonads	1241	Type I	CAGTATTAC	X	X
*C. sapidus* Atlantic Blue Crab aCV (I0056)	*Callinectes sapidus*	Gonads	1876	Type I	CAGTATTAC	X	X
*P. intermedius* Brackish Grass Shrimp aCV (I0059)	*Palaemonetes intermedius*	Whole organism	2293	Type I	CAGTATTAC	X^∗^	X
*F. duorarum* Pink Shrimp aCV (I0066)	*Farfantepenaeus duorarum*	Whole organism	1799	Type I	CAGTATTAC	X	X
*F. duorarum* Pink Shrimp aCV (I0069)	*Farfantepenaeus duorarum*	Whole organism	1966	Type I	CAGTATTAC	X^∗^	X
Marine Snail aCV (I0084)	Marine Snail	Whole organism	2305	Type I	TAGTATTAC	X^∗^	X
Hermit Crab aCV (I0085A4)	Hermit Crab	Abdomen	2291	Type I	TAGTATTAC	X^∗^	X
Hermit Crab aCV (I0085A5)	Hermit Crab	Abdomen	2291	Type I	TAGTATTAC	X^∗^	X
Hermit Crab aCG (I0085b)	Hermit Crab	Abdomen	1063	Type VII	CAGTATTAC		X
Fiddler Crab aCV (I0086a)	Fiddler Crab	Gonads and claw muscle	1635	Type II	GATTATTAC	X	X
Fiddler Crab aCV (I0086b)	Fiddler Crab	Gonads and claw muscle	1511	Type V	AAGTATTAC	X	X
*P. kadiakensis* Mississippi Grass Shrimp aCV (I0099)	*Palaemonetes kadiakensis*	Whole organism	1895	N/A	None	X^∗^	X
*Gammarus* sp. Amphipod aCV (I0153)	*Gammarus* sp.	Whole organism	1999	Type I	TAGTATTAC	X^∗^	X
*Mytilus* sp. Clam aCV (I0169)	*Mytilus* sp.	Whole organism	1894	Type I	TAGTATTAC	X	X
*Calanoida* sp. Copepod aCV (I0298)	*Calanoida* sp.	Whole organism	2469	Type II	TAGTATTAC	X	X
*A. melana* Sponge aCG (I0307)	*Artemia melana*	Tissue segment	1826	Type VII	TAGTATTAC		X
*P. pacifica* Coral aCV (I0345)	*Primnoa pacifica*	Polyps	1240	N/A	None	X^∗^	X
*P. placomus* Coral aCV (I0351)	*Paramuricea placomus*	Polyps	2292	Type II	TAGTATTAC	X^∗^	X
*S. brevirostris* Brown Rock Shrimp aCV (I0722)	*Sicyonia brevirostris*	Gonads	1600	Type V	TAATATTAC^∗^	X	X

*^1^Genome names contain abbreviation aCV for associated circular virus or aCG for associated circular genome. ID within parentheses corresponds to ID used throughout the paper.*

*^2^Nonanucleotide motif sequences that were not identified within a stem-loop structure are denoted with an asterisk (^∗^).*

*^3^Non-Rep encoding ORFs were identified as putative capsid proteins based on BLAST results. However, many non-Rep-encoding ORFs did not exhibit any significant matches (marked with an asterisk^∗^)*.

DNA extracts were amplified through RCA using the illustra TempliPhi Amplification kit (GE Healthcare) to enrich for small circular templates (Kim et al., 2008; Kim and Bae, 2011). RCA-amplified DNA was digested with a suite of FastDigest restriction enzymes (Life Technologies; BamHI, EcoRV, PdmI, HindIII, KpnI, PstI, XhoI, SmaI, BgiII, EcoRI, XbaI, and NcoI) following manufacturer’s instructions in separate reactions to obtain complete, unit-length genomes for downstream cloning and sequencing. Restriction enzyme digested products were resolved on an agarose gel and bands ranging in size from 1000 to 4000 bp were excised and cleaned using the Zymoclean Gel DNA Recovery Kit (Zymo Research). Products resulting from blunt-cutting enzyme digestions were cloned using the CloneJET PCR Cloning kit (Life Technologies), whereas products containing sticky ends were cloned using pGEM-3Zf(+) vectors (Promega) pre-digested with the appropriate enzyme. All clones were commercially Sanger sequenced using vector primers and genomes exhibiting significant similarities to eukaryotic CRESS-DNA viruses were completed through primer walking.

### Genome Annotation

Genomes were assembled using Sequencher 4.1.4 (Gene Codes Corporation). Putative ORFs >100 amino acids were identified and annotated using SeqBuilder version 11.2.1 (Lasergene). Partial genes or genes that seemed interrupted were analyzed for potential introns using GENSCAN (Burge and Karlin, 1997). The potential origin of replication (*ori*) for each genome was identified by locating a canonical nonanucleotide motif (NANTATTAC; Rosario et al., 2012a) and confirming predicted stem-loop structures using Mfold with constraints applied to prevent hairpin formation within the nonanucleotide motif and a folding temperature set at 17°C (Zuker, 2003). Final annotated genomes have been deposited to GenBank with accession numbers KR528543–KR528569.

### Database Sequences and Sequence Analysis

To conduct sequence comparisons, members of the *Circovirus* genus, as well as complete eukaryotic CRESS-DNA viral genomes obtained from environmental samples or individual organisms through either metagenomic sequencing or degenerate PCR (herein referred to as “metagenomic CRESS-DNA viruses”) were retrieved from GenBank. Since the Rep is the only conserved protein among CRESS-DNA viruses (Ilyina and Koonin, 1992; Rosario et al., 2012a) this protein was used to compare the different genomes. Rep pairwise identities were calculated using SDT v1.2 (Muhire et al., 2014) and summarized using heat maps generated in R (R Core Team, 2014). A maximum likelihood (ML) phylogenetic tree based on Rep amino acid sequences was also constructed. For this purpose, alignments were performed in MEGA 6.06 (Tamura et al., 2013) using the MUSCLE algorithm (Edgar, 2004) and manually edited. Sequences were inspected for the presence of conserved amino acid motifs that have been shown to play a role in rolling circle replication (RCR) of eukaryotic CRESS-DNA viruses, including three RCR and three superfamily 3 (SF3) helicase motifs (Gorbalenya et al., 1990; Ilyina and Koonin, 1992; Gorbalenya and Koonin, 1993; Rosario et al., 2012a). Although all the recently reported CRESS-DNA viruses are included in the heatmap, only sequences exhibiting all six motifs are included in the phylogenetic analysis. In addition, divergent regions that were poorly aligned, as shown by a high percentage of gaps, were removed from the alignment (Supplementary Data Sheet 1). Since the *Nanoviridae* and *Geminiviridae* are also CRESS-DNA viral families that are evolutionarily related to the *Circoviridae* (Ilyina and Koonin, 1992; Rosario et al., 2012a), select representatives of these families were included in the phylogenetic analysis. The ML phylogenetic tree was inferred using PHYML (Guindon et al., 2010) implementing the best substitution model (rtRev+I+G+F; Dimmic et al., 2002) according to ProtTest (Abascal et al., 2005). Branch support was assessed using the approximate likelihood ratio test (aLRT) SH-like method (Anisimova and Gascuel, 2006).

### Intrinsically Disordered Region (IDR) Analysis of Putative Capsid Proteins

To determine if the non-Rep-encoding ORFs from the CRESS-DNA viral genomes presented here (*n* = 25), circoviruses (*n* = 15), and metagenomic CRESS-DNA viruses (*n* = 259; including 37 cycloviruses) represent putative Caps, these proteins were evaluated for IDRs. Disordered protein regions were predicted using the DisProt VL3 disorder predictor (Obradovic et al., 2003; Sickmeier et al., 2007). This artificial neural network utilizes an ensemble of feed forward neural networks with 20 attributes (18 amino acid frequencies, average flexibility, and sequence complexity; Obradovic et al., 2003). Disorder disposition scores above a 0.5 threshold indicate intrinsic disorder. Counts and statistical analysis for the fraction of disorder- and order-promoting amino acid residues was conducted using R with the “seqinr” package (Charif and Lobry, 2007).

## Results

**FIGURE 1:**
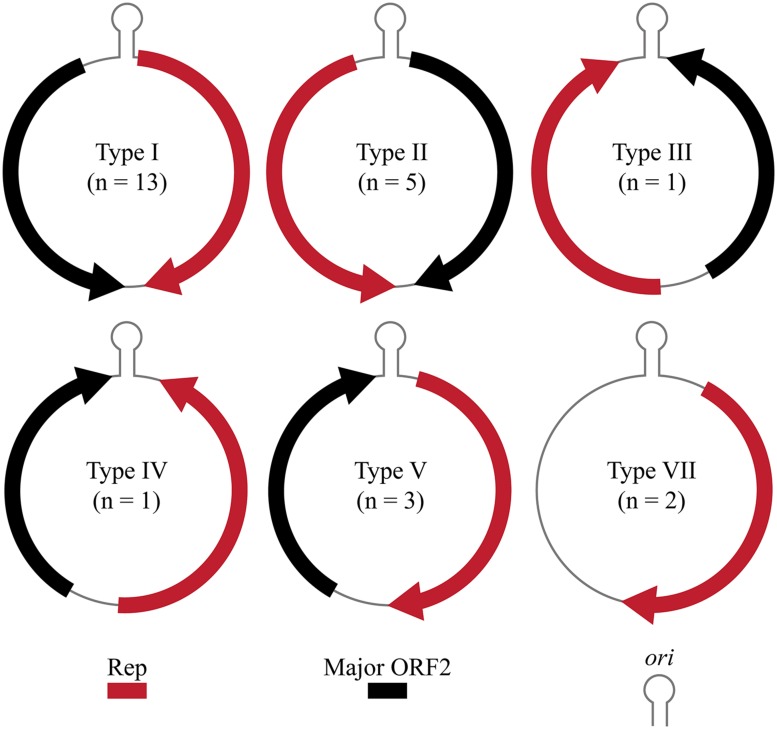
**Genome types of novel CRESS-DNA genomes identified in this study (Rosario et al., 2012a).** Genome schematics illustrate a major ORF encoding the replication initiator protein (Rep), putative origin of replication (*ori)* marked by stem-loop structure, and a second major ORF.

A total of 27 CRESS-DNA genomes were recovered from 21 marine invertebrates (**Table 1**). Most of the recovered genomes (66.7%) were identified from *Crustacea*, mainly from the order *Decapoda*. Recovered genomes ranged in size from 1063 to 2469 nt and exhibited a variety of genome architectures. Of the 27 genomes identified, 23 exhibited a common putative *ori* marked by a conserved nonanucleotide motif (NANTATTAC) at the apex of a predicted stem-loop structure (**Table 1**). The remaining four genomes lacked a stem-loop structure (*n* = 2) or a stem-loop structure and a nonanucleotide motif (*n* = 2). Genomes lacking the canonical nonanucleotide motif could not be assigned to any genome type; therefore only 25 genomes were assigned to genomic architecture types previously described by Rosario et al. (2012a) (**Figure 1**). The predominant genomic architecture observed was Type I (*n* = 13), which is typical of members of the *Circovirus* genus. However, other genomic architectures were observed including Types II (*n* = 5), III (*n* = 1), IV (*n* = 1), V (*n* = 3), and VII (*n* = 2) (**Figure 1**). It is important to note that genomes exhibiting a Type VII genome architecture only exhibit a single major ORF encoding a Rep. This type of architecture is observed in genomic components of multipartite viruses from the *Nanoviridae* family and satellite DNA molecules that require helper viruses for encapsidation (Gronenborn, 2004; Briddon and Stanley, 2006). Therefore genomes exhibiting only a single major ORF may represent partial genomes of multipartite viruses or non-viral mobile genetic elements such as plasmids (Rosario et al., 2012a).

The majority of the CRESS-DNA viruses detected in marine invertebrates were most similar to viral sequences identified through metagenomic surveys of marine samples (Supplementary Table S1). However, one of genomes, *Lytechinus variegatus* variable sea urchin associated circular virus_I0021, was most similar to plant viruses from the *Geminiviridae* family. Most of the viral genomes had database similarities for the Rep; except for *Sicyonia brevirostris* brown rock shrimp associated circular virus_I0722, which only had similarities for the putative Cap (Supplementary Table S1). Similar to several previously described CRESS-DNA viruses (Li et al., 2010a; Rosario et al., 2012b; van den Brand et al., 2012; Sikorski et al., 2013b; Du et al., 2014; Ng et al., 2014; Dayaram et al., 2015a,b; Kraberger et al., 2015), three viral genomes (*Artemia melana* sponge associated circular virus_I0307, *Didemnum* sp. sea squirt associated circular virus_I0026_A7, and *Palaemonetes kadiakensis* Mississippi grass shrimp associated circular virus_I0099) exhibited Reps interrupted by introns (Supplementary Table S1).

**FIGURE 2:**
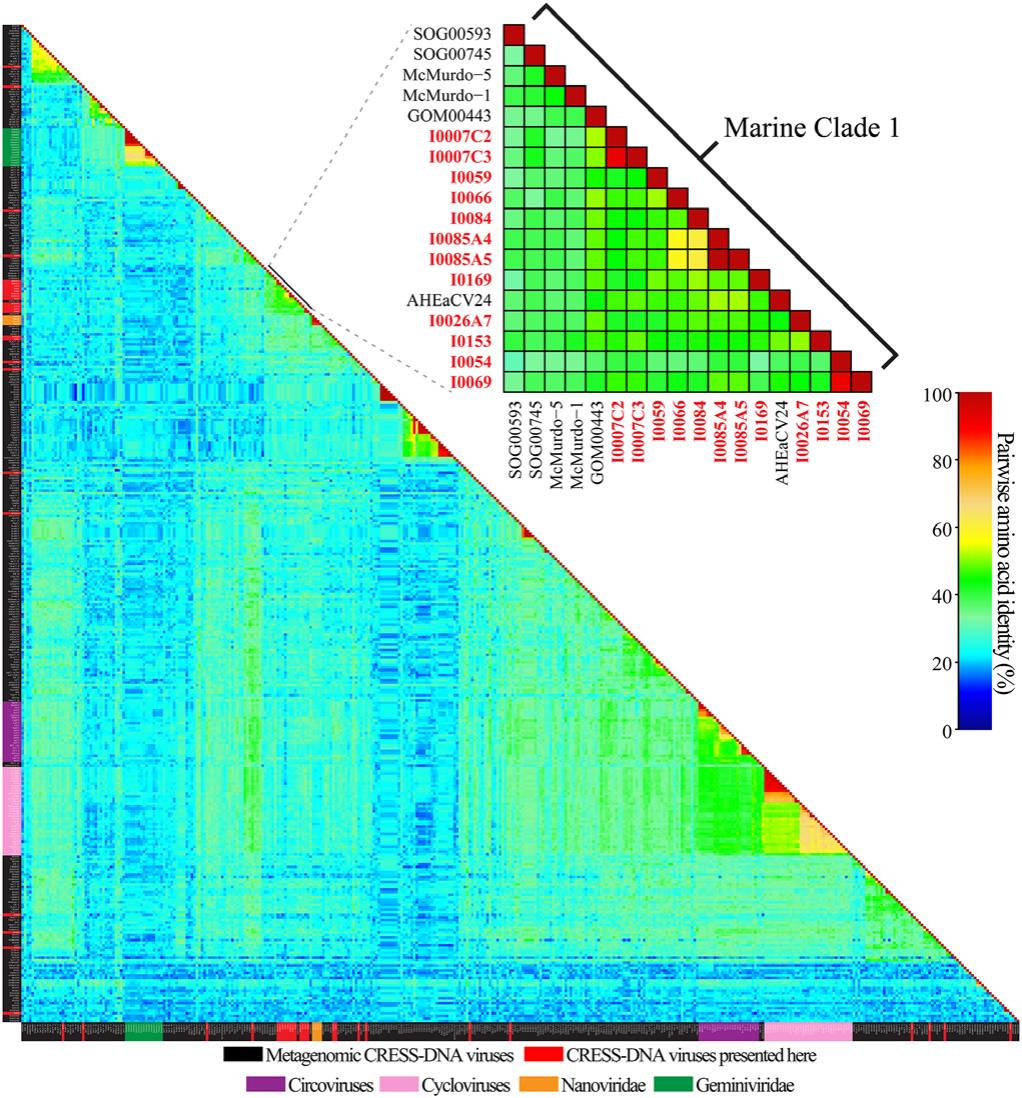
**Graphical representation of pairwise amino acid identities of the replication initiator proteins (Rep) from CRESS-DNA genomes from this study, metagenomic CRESS-DNA viruses, cycloviruses, circoviruses, and select members of the *Nanoviridae and Geminiviridae* families.** Reps identified from this study within the Marine Clade 1 are in red font. Description of acronyms and the matrix used to generate the heatmap can be found in Supplementary Tables S2 and S3, respectively.

**FIGURE 3:**
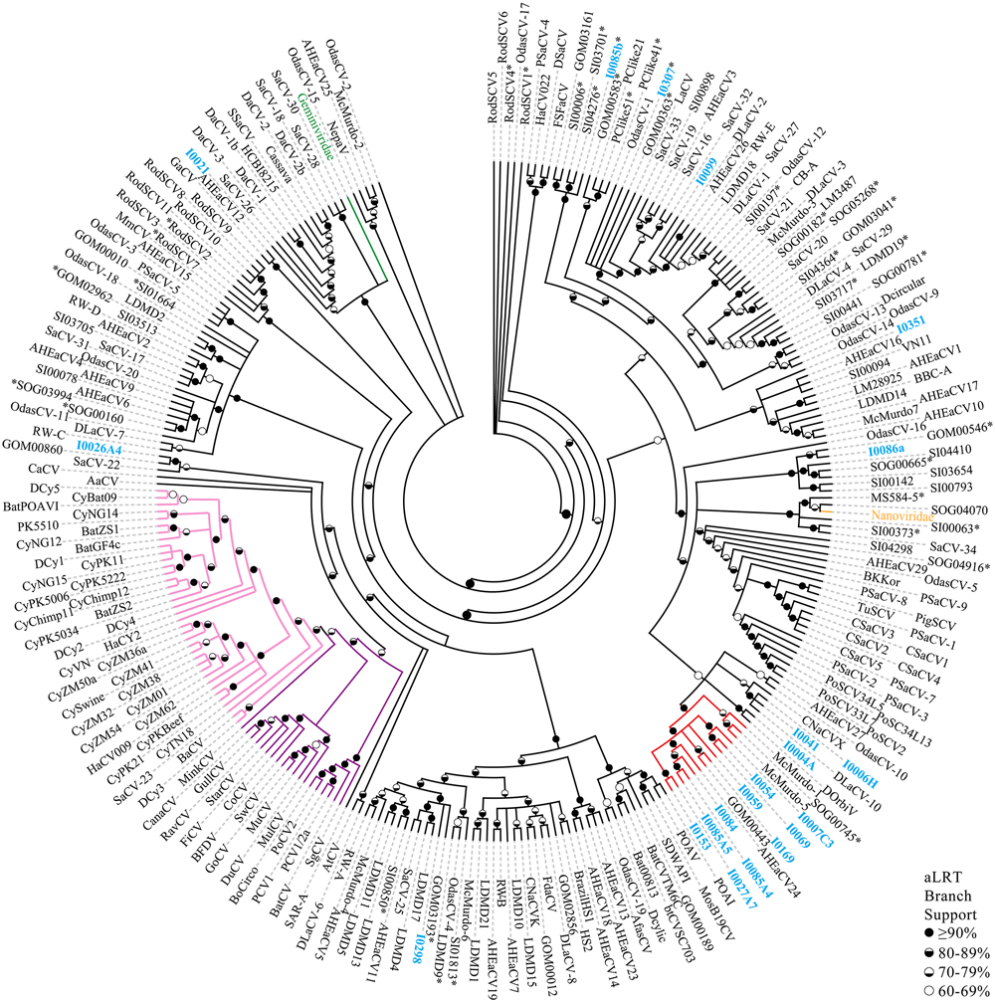
**Multifurcation maximum likelihood phylogenetic reconstruction based on the Reps of CRESS-DNA genomes recovered here, metagenomic CRESS-DNA viruses, cycloviruses, circoviruses, and representative members of the *Nanoviridae and Geminiviridae* families.** Reps obtained from CRESS-DNA genomes obtained in this study are highlighted in blue font. Branches are colored for the different CRESS-DNA viral groups including the Marine Clade 1 (red), circoviruses (purple), cycloviruses (pink), nanoviruses (orange), and geminiviruses (green). Representative nanoviruses (*n* = 4) and geminiviruses (*n* = 15) have been condensed into their family names. Reps from genomes exhibiting a single ORF are highlighted using an asterisk (^∗^). Branches with less than 60% aLRT branch support have been collapsed. Description of acronyms used can be found in Supplementary Table S4.

Pairwise identities indicate that the CRESS-DNA viruses detected in marine invertebrates share less than 60.1% sequence identity (average sequence identity = 26.04%) with previously identified Reps from CRESS-DNA viruses in GenBank, indicating that these viruses represent novel species (**Figure 2**). Twenty-one of the 27 recovered Reps contained all six conserved RCR and helicase motifs (see Materials and Methods) and were used for phylogenetic analysis. Analysis of these Reps with representative CRESS-DNA viral Reps from GenBank, including available metagenomic CRESS-DNA viral Reps, show that most of the sequences from marine invertebrate associated viruses detected here are more closely related to circo-like viruses recovered through metagenomic surveys of the marine environment than to previously defined CRESS-DNA viral groups (**Figure 3**). Eleven of the 21 Reps from marine invertebrate associated viruses do not form distinct clusters with each other or any known sequences (**Figure 3**). However, ten of the Reps form a well-supported clade that also includes sequences detected in the Gulf of Mexico (GOM00443; JX904231.1), Straight of Georgia (JX904106.1), McMurdo Ice Shelf (YP_009047125.1; YP_009047137.1), and a semi-enclosed shallow estuary (Avon-Heathcote Estuary associated circular virus 24; AJP36460.1). Pairwise identity scores indicate that all members of this clade, named Marine Clade 1 for the purposes of this study, share more than 32.7% identity, with an average pairwise identity score of 47.2% (**Figure 2**). Members of the Marine Clade 1 seem to be more closely related to members of the *Nanoviridae* (31.95% average pairwise identity) than any other known CRESS-DNA viral group; however, members of this clade exhibit different genomic architectures compared to these plant viruses. CRESS-DNA viral genomes from the Marine Clade 1 encode two major ORFs in an ambisense organization (i.e., Type I architecture), which is similar to members of the *Circoviridae*, rather than the single ORF, Type VII genome organization observed in genomic components from the *Nanoviridae*.

### Capsid Analysis

Only half of the CRESS-DNA viral genomes described here contained an ORF that had significant BLASTX matches (e-value < 0.001; amino acid identities ranging from 26–54%) to proteins annotated as putative Caps in GenBank (**Table 1**). Furthermore, most of the matches in the database were to putative CRESS-DNA viral Caps detected through metagenomic surveys, which are not supported by biochemical data and have not necessarily been well curated. Therefore, alternative methods were explored to investigate non-Rep-encoding ORFs (i.e., putative Caps) found in CRESS-DNA viral genomes.

The majority of metagenomic CRESS-DNA viruses reported from marine invertebrates in this study and in GenBank are most similar to previously described circoviruses. Therefore, the predicted IDP profiles of well-characterized members of the *Circovirus* genus were examined in an effort to identify conserved patterns in structural proteins encoded by these viruses. These circovirus IDP profiles were then compared against profiles observed in cycloviruses (the proposed sister group to the circoviruses, which exhibit conserved features and share high identities with circoviruses) and other metagenomic CRESS-DNA viruses.

**FIGURE 4:**
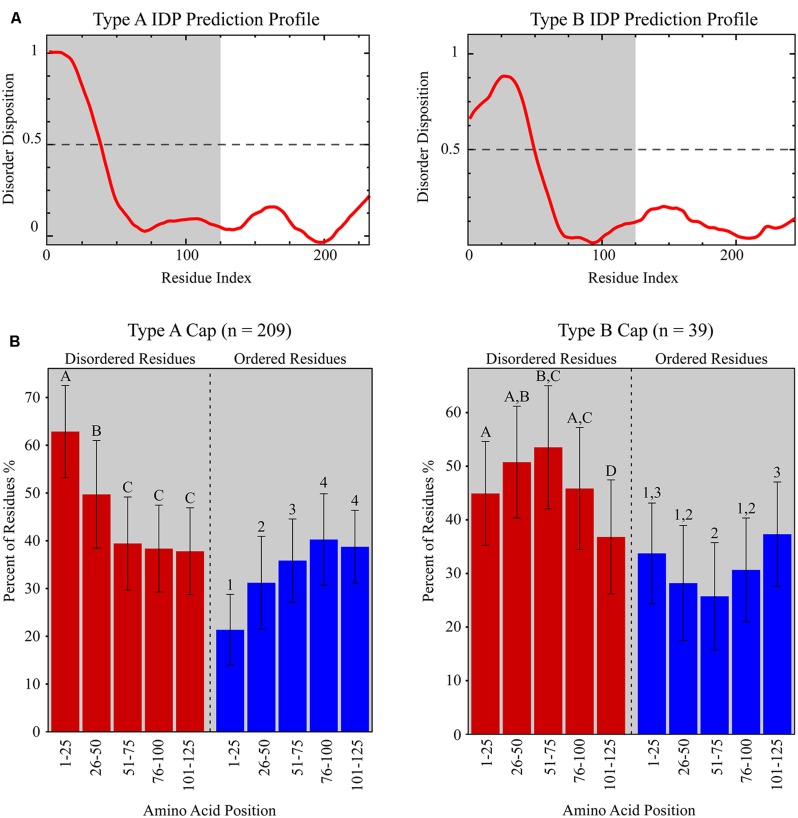
**(A)** Representative IDP prediction profiles for Type A and Type B capsid proteins (Caps) from the Disprot VL3 predictor. Type A and Type B IDP prediction profiles are based on the Porcine circovirus 2 Cap (NP_937957.1) and the Beak and feather disease virus Cap (NP_047277.1), respectively. The grey shaded area represents the amino acid residue interval used in **(B)**. **(B)** Graphs showing the fraction of predicted disordered (red bars) and ordered (blue bars) residues within discrete amino acid intervals for Type A and Type B Caps identified from all CRESS-DNA viral genomes analyzed in this study. Significantly different amino acid intervals for each Cap type are distinguished using letters (“A”, “B”, “C”, “D” for statistics based on percentage of predicted disordered residues) or numbers (“1”, “2”, “3”, “4” for statistics based on percentage of predicted ordered residues; ANOVA with *post hoc* Tukey’s HSD; *p* < 0.05). Note that the percentage of predicted disordered and ordered residues does not add to 100% due to the presence of residues that are not considered either disordered or ordered (i.e., H, M, T, and D).

The DisProt VL3 disorder prediction analysis revealed that Caps encoded by members of the *Circovirus* genus (*n* = 15) exhibit one of two protein disorder profiles, distinguished here as Type A or Type B, based on the first 125 amino acids of these proteins (**Figure 4A**). Type A Caps exhibit IDP profiles that are predicted to have the highest degree of disorder closest to the N-terminus (i.e., amino acid residues 1–50) before the profile tapers to a structured region with variable predicted disorder. Type A Caps exhibit significant enrichment for amino acid residues that promote disorder (R, K, E, P, S, Q, and A) within the first 50 residues relative to amino acid residues 51–125 (ANOVA with *post hoc* Tukey’s HSD; *p* < 0.05) and a depletion of order promoting amino acid residues (W, C, F, I, Y, V, L, and N) within the first 25 residues relative to amino acid residues 26–125 (ANOVA with *post hoc* Tukey’s HSD; *p* < 0.05; **Figure 4B**). On the other hand, Type B Caps exhibit IDP profiles that peak in predicted disorder between amino acid residues 26–75. Type B Caps show an enrichment of disorder promoting residues between residue positions 26 through 75, whereas there is a depletion of predicted order promoting residues in this region compared to residues 1–25 and 76–125 (**Figure 4B**). Beyond 125 amino acids, IDP profiles exhibited more structured regions for both Types A and B Caps, with no distinguishable predicted disorder pattern (**Figure 4A**).

The overwhelming majority of Caps from the *Circovirus* genus (86.7%) exhibited Type A IDP profiles; however, two avian circoviruses, Finch circovirus (YP_803551.1) and Beak and feather disease virus (NP_047277.1), had Type B IDP profiles (**Table 2** and Supplementary Table S5). Similarly, 97.3% of cyclovirus putative Caps (*n* = 37) exhibited Type A IDP profiles. Comparison of IDP profiles showed that a majority of metagenomic CRESS-DNA viruses also contained patterns of increased predicted disorder at the N-terminus of the putative Cap, consistent with the *Circoviridae*. Interestingly, Type B IDP profiles were more prevalent among putative Caps from metagenomic CRESS-DNA viral genomes in GenBank (10.8%; *n* = 222) and the novel genomes reported in this study (56%; *n* = 25). Notably, 7 of the 10 viruses found in the Marine Clade 1 described here exhibit Type B Caps. Among the total 299 CRESS-DNA genome sequences analyzed, most putative Caps exhibit Type A IDP profiles (69.9%), followed by Type B (13%). Notably, most of the putative Caps lacking a significant match in the database exhibited one these profiles.

**Table 2:** Intrinsically disordered protein (IDP) profile types identified in non-Rep encoding ORFs of CRESS-DNA viruses.

Group	Total sequences	IDP Cap type	
		Type A	Type B	No type
Circoviruses	15	86.7%	13.3%	0.0%
Cycloviruses	37	97.3%	0.0%	2.7%
Metagenomic CRESS-DNA viruses	222	67.6%	10.8%	21.6%
This study	25	40.0%	56.0%	4.0%
**Total**	299	69.9%	13.0%	17.1%

## Discussion

Metagenomic studies have revealed a prodigious amount of diversity in eukaryotic CRESS-DNA viruses in the marine environment (Rosario et al., 2009a; Rosario and Breitbart, 2011; Labonte and Suttle, 2013; McDaniel et al., 2014). However, few studies have isolated these viruses directly from organisms. Building upon recent studies suggesting that CRESS-DNA viruses are associated with marine invertebrates (Dunlap et al., 2013; Hewson et al., 2013a,b; Ng et al., 2013; Pham et al., 2014; Soffer et al., 2014; Dayaram et al., 2015a), this study investigated a variety of marine invertebrates, including under sampled taxa, for the presence of these viruses. Viral genomes presented here were primarily recovered from *Crustacea*, suggesting that this subphylum harbors a rich diversity of CRESS-DNA viruses. This is consistent with previous research that identified CRESS-DNA viruses in copepods (Dunlap et al., 2013), which are the most abundant members of mesozooplankton (Kleppel et al., 1996), as well as different species of shrimp (Ng et al., 2013; Pham et al., 2014), which comprise some of the world’s most important food sources (Goss et al., 2000; Paezosuna, 2003). In addition, this is the first study to report viruses associated with marine snails, anemones, sea squirts, and several crab species. Although a definitive host for these viruses cannot be assigned with the present data, this study reveals the need for further examination of viruses associated with common marine invertebrates and experiments to determine their potential impact, if any, on the ecology of these organisms. The grouping of the invertebrate-associated CRESS-DNA viruses reported here with metagenomic CRESS-DNA viruses implies that marine invertebrates may serve as hosts for many of the sequences obtained from marine environments.

The marine invertebrate associated CRESS-DNA viruses identified here are only distantly related to known members of the *Circoviridae* and may represent novel groups. Approximately one third of the novel sequences reported here belong to the Marine Clade 1, whose members share an average pairwise identity of 47.2%. Members of this viral clade share an average pairwise identity score of 27.5% with members of the *Circoviridae*, whose members (genus *Circovirus* and proposed genus Cyclovirus) share 48.9% average pairwise identity. Although members of the Marine Clade 1 share slightly higher average pairwise identity with the *Nanoviridae* (31.2%), their genome architecture is clearly distinct from these plant-infecting viruses. Therefore, genomic architectures and comparative Rep analyses suggest that members of the Marine Clade 1 may represent a novel CRESS-DNA viral family.

The highly conserved Rep enables its straightforward identification through similarity-based searches; however, there is currently no reliable method for characterizing highly divergent putative Caps for metagenomic CRESS-DNA viruses. Since many of the novel metagenomic CRESS-DNA viruses are most similar to members of the *Circoviridae*, which only contain two major ORFs encoding a Rep and Cap, the putative Cap is often assigned simply based on the conserved genome architectures exhibited by this group.

This study investigated the IDP profiles of all available circo-like CRESS-DNA viruses to evaluate if putative Caps exhibit conserved patterns that could be used to identify this structural protein even in the absence of significant similarities in the database. The Cap of Porcine circovirus 2 represents a Type A IDP profile and that of Beak and feather disease virus represents a Type B IDP profile. Since the non-Rep-encoding ORF for both of these circoviruses have been shown to be structural (Nawagitgul et al., 2000; Patterson et al., 2013), this provides evidence that both the Type A and Type B IDP profiles represent a Cap. These Cap IDP profiles may be driven by the arginine and/or lysine rich region at the N-terminus of the Cap (Niagro et al., 1998), as both of these amino acids are considered disorder-promoting residues by the DisProt VL3 neural network. In addition to characterizing IDP profiles of circo-like CRESS-DNA viruses, analysis of select *Geminiviridae* and *Nanoviridae* Caps demonstrated that these viruses also exhibit Type A and Type B IDP profiles (Supplementary Table S5). Although further research into these plant virus families is needed, these findings suggest that the IDP patterns identified here may be conserved across Caps from the different families of eukaryotic CRESS-DNA viruses.

Thirteen of the eukaryotic CRESS-DNA viruses presented here had a non-Rep-encoding ORF without any database similarities, which were characterized as a putative Cap based on IDP profiles. Likewise, hypothetical proteins from 32 metagenomic CRESS-DNA viruses were identified as putative Caps using this method (Supplementary Table S5). While the Caps in the database were dominated by Type A IDP profiles, the majority of the new marine invertebrate associated genomes presented here exhibited Type B IDP profiles. In addition, 50 of the CRESS-DNA genomes analyzed here (17.1%; *n* = 299), including the *Primnoa pacifica* coral associated circular virus I0345 identified here, contained a non-Rep-encoding ORF that did not exhibit either the Type A or Type B profile. While it is possible that other IDP profiles representative of novel Caps exist, caution should be used in annotating these ORFs as putative Caps without supporting evidence. Finally, while examining metagenomic sequences annotated as CRESS-DNA viruses in GenBank, numerous genomes were identified that only contained a single ORF, which encoded a Rep. These sequences (Supplementary Table S5), along with the two Type VII genomes found in this study, most likely represent partial viral genomes [i.e., a single component of a multipartite virus (Gutierrez, 1999; Gronenborn, 2004)], satellite DNA molecules (Briddon and Stanley, 2006), or non-viral mobile genetic elements (Rosario et al., 2012a). Genomes exhibiting a single ORF cannot be distinguished phylogenetically from complete viral genomes based on the Rep (**Figure 3**). Therefore, it is important to investigate complete genomes of CRESS-DNA viruses rather than partial sequences.

The IDP analysis has interesting implications for understanding the evolutionary pressures acting upon the Rep and Cap of CRESS-DNA viruses, which include the smallest known eukaryotic viral pathogens. Small viruses exhibit a higher proportion of predicted disordered residues than larger viruses and may exploit IDRs to encode multifunctional proteins (Xue et al., 2012, 2014; Pushker et al., 2013). Rep proteins encoded by CRESS-DNA viruses exhibited low disposition for predicted disorder promoting amino acid residues or an inconsistency in predicted disorder patterns (data not shown), while the Caps consistently exhibited profiles with increased predicted disorder at the N-terminus, suggesting that the high proportion of predicted disordered regions in these small viruses may be driven by the Cap. IDRs have a tendency to evolve more rapidly than structured regions (Brown et al., 2002, 2011; Chen et al., 2006; Bellay et al., 2011; Nilsson et al., 2011; van der Lee et al., 2014); consequently, IDRs may hinder our ability to perform phylogenetic reconstructions based on the Cap. Although we are unable to perform reliable Cap alignments, the ability to classify these proteins within CRESS-DNA virus genomes due to conserved predicted disorder profiles reveals that these viruses exhibit regions in which disorder is conserved despite rapidly evolving amino acids (i.e., flexible disorder; van der Lee et al., 2014).

Although the functional significance of predicted IDP profiles detected in this study has yet to be determined, the identification of conserved IDP profiles may prove useful to identify divergent structural proteins encoded by CRESS-DNA viruses. The identification of a given IDP profile (Type A or B) for a putative ORF in a genomic context may allow the recognition of novel CRESS-DNA viral structural proteins that cannot be identified by standard BLAST searches. The IDP profile analysis needs to be complemented by other genomic features that are characteristic of CRESS-DNA viruses, including the presence of a Rep exhibiting RCR and helicase motifs and a putative *ori* marked by a conserved nonanucleotide motif (NANTATTAC) at the apex of a stem-loop structure. Future work needs to evaluate if the high proportion of IDRs observed in CRESS-DNA viruses and other small viruses is indeed mainly driven by structural proteins. If this observation is validated, IDP profile analysis of hypothetical proteins may provide a reliable tool to identify structural proteins encoded by small viruses.

## Conflict of Interest Statement

The authors declare that the research was conducted in the absence of any commercial or financial relationships that could be construed as a potential conflict of interest.
