# Supplementary material for: Novel circular single-stranded DNA viruses identified in marine invertebrates reveal high sequence diversity and consistent predicted intrinsic disorder patterns within putative structural proteins
Source: Front Microbiol. 2015 Jul 10;6:696. doi: 10.3389/fmicb.2015.00696 (PMC4498126; doi:10.3389/fmicb.2015.00696)
Supplement: Supplementary file 2 [file Table_2.DOCX]

**Supplementary Table S2.** Rep sequences used for pairwise amino acid identities, their corresponding GenBank accession numbers, a brief descriptive title, and acronyms used for Figure 2.

| Accession | Descriptive Title | Acronym |
| --- | --- | --- |
| JX904075.1 | Uncultured marine virus clone SOG00160 | SOG00160 |
| JX904076.1 | Uncultured marine virus clone SOG00164 | SOG00164 |
| JX904077.1 | Uncultured marine virus clone SOG00182 | SOG00182 |
| JX904094.1 | Uncultured marine virus clone SOG00593 | SOG00593 |
| JX904100.1 | Uncultured marine virus clone SOG00665 | SOG00665 |
| JX904106.1 | Uncultured marine virus clone SOG00745 | SOG00745 |
| JX904107.1 | Uncultured marine virus clone SOG00781 | SOG00781 |
| JX904108.1 | Uncultured marine virus clone SOG00824 | SOG00824 |
| JX904109.1 | Uncultured marine virus clone SOG00852 | SOG00852 |
| JX904121.1 | Uncultured marine virus clone SOG01527 | SOG01527 |
| JX904139.1 | Uncultured marine virus clone SOG03994 | SOG03994 |
| JX904144.1 | Uncultured marine virus clone SOG04070 | SOG04070 |
| JX904151.1 | Uncultured marine virus clone SOG04311 | SOG04311 |
| JX904155.1 | Uncultured marine virus clone SOG04508 | SOG04508 |
| JX904169.1 | Uncultured marine virus clone SOG04916 | SOG04916 |
| JX904185.1 | Uncultured marine virus clone SOG05268 | SOG05268 |
| JX904191.1 | Uncultured marine virus clone GOM00010 | GOM00010 |
| JX904192.1 | Uncultured marine virus clone GOM00012 | GOM00012 |
| JX904207.1 | Uncultured marine virus clone GOM00189 | GOM00189 |
| JX904221.1 | Uncultured marine virus clone GOM00363 | GOM00363 |
| JX904231.1 | Uncultured marine virus clone GOM00443 | GOM00443 |
| JX904245.1 | Uncultured marine virus clone GOM00546 | GOM00546 |
| JX904250.1 | Uncultured marine virus clone GOM00583 | GOM00583 |
| JX904278.1 | Uncultured marine virus clone GOM00857 | GOM00857 |
| JX904279.1 | Uncultured marine virus clone GOM00860 | GOM00860 |
| JX904312.1 | Uncultured marine virus clone GOM02856 | GOM02856 |
| JX904333.1 | Uncultured marine virus clone GOM02962 | GOM02962 |
| JX904344.1 | Uncultured marine virus clone GOM03041 | GOM03041 |
| JX904368.1 | Uncultured marine virus clone GOM03161 | GOM03161 |
| JX904373.1 | Uncultured marine virus clone GOM03175 | GOM03175 |
| JX904377.1 | Uncultured marine virus clone GOM03193 | GOM03193 |
| JX904394.1 | Uncultured marine virus clone SI00003 | SI00003 |
| JX904395.1 | Uncultured marine virus clone SI00006 | SI00006 |
| JX904401.1 | Uncultured marine virus clone SI00063 | SI00063 |
| JX904407.1 | Uncultured marine virus clone SI00078 | SI00078 |
| JX904412.1 | Uncultured marine virus clone SI00094 | SI00094 |
| JX904416.1 | Uncultured marine virus clone SI00142 | SI00142 |
| JX904420.1 | Uncultured marine virus clone SI00197 | SI00197 |
| JX904427.1 | Uncultured marine virus clone SI00349 | SI00349 |
| JX904431.1 | Uncultured marine virus clone SI00373 | SI00373 |
| JX904439.1 | Uncultured marine virus clone SI00441 | SI00441 |
| JX904469.1 | Uncultured marine virus clone SI00793 | SI00793 |
| JX904473.1 | Uncultured marine virus clone SI00850 | SI00850 |
| JX904478.1 | Uncultured marine virus clone SI00898 | SI00898 |
| JX904518.1 | Uncultured marine virus clone SI01664 | SI01664 |
| JX904523.1 | Uncultured marine virus clone SI01813 | SI01813 |
| JX904541.1 | Uncultured marine virus clone SI03513 | SI03513 |
| JX904548.1 | Uncultured marine virus clone SI03654 | SI03654 |
| JX904559.1 | Uncultured marine virus clone SI03701 | SI03701 |
| JX904561.1 | Uncultured marine virus clone SI03705 | SI03705 |
| JX904562.1 | Uncultured marine virus clone SI03717 | SI03717 |
| JX904566.1 | Uncultured marine virus clone SI03747 | SI03747 |
| JX904581.1 | Uncultured marine virus clone SI03931 | SI03931 |
| JX904605.1 | Uncultured marine virus clone SI04276 | SI04276 |
| JX904614.1 | Uncultured marine virus clone SI04298 | SI04298 |
| JX904629.1 | Uncultured marine virus clone SI04364 | SI04364 |
| JX904640.1 | Uncultured marine virus clone SI04410 | SI04410 |
| JX904647.1 | Uncultured marine virus clone SI04458 | SI04458 |
| JX904674.1 | Uncultured marine virus clone SI04666 | SI04666 |
| JX904655.1 | Uncultured marine virus clone SI04504 | SI04504 |
| JX904639.1 | Uncultured marine virus clone SI04406 | SI04406 |
| JX904575.1 | Uncultured marine virus clone SI03890 | SI03890 |
| JX904511.1 | Uncultured marine virus clone SI01524 | SI01524 |
| JX904424.1 | Uncultured marine virus clone SI00292 | SI00292 |
| JX904415.1 | Uncultured marine virus clone SI00102 | SI00102 |
| JX904403.1 | Uncultured marine virus clone SI00067 | SI00067 |
| JX904378.1 | Uncultured marine virus clone GOM03194 | GOM03194 |
| JX904359.1 | Uncultured marine virus clone GOM03116 | GOM03116 |
| JX904346.1 | Uncultured marine virus clone GOM03098 | GOM03098 |
| JX904147.1 | Uncultured marine virus clone SOG04106 | SOG04106 |
| JX904118.1 | Uncultured marine virus clone SOG01184 | SOG01184 |
| JX904101.1 | Uncultured marine virus clone SOG00690 | SOG00690 |
| JX904555.1 | Uncultured marine virus clone SI03691 | SI03691 |
| JX904472.1 | Uncultured marine virus clone SI00841 | SI00841 |
| JX904301.1 | Uncultured marine virus clone GOM01500 | GOM01500 |
| JX904172.1 | Uncultured marine virus clone SOG04928 | SOG04928 |
| JX904134.1 | Uncultured marine virus clone SOG03823 | SOG03823 |
| JX904099.1 | Uncultured marine virus clone SOG00662 | SOG00662 |
| JX904098.1 | Uncultured marine virus clone SOG00640 | SOG00640 |
| JX904092.1 | Uncultured marine virus clone SOG00568 | SOG00568 |
| YP_164517.1 | Muscovy duck circovirus | MuDCV |
| NP_877978.1 | Mulard duck circovirus | MulDCV |
| YP_009109623.1 | PoCircolike virus 51 | PClike51 |
| YP_009109620.1 | PoCircolike virus 41 | PClike41 |
| YP_009109615.1 | PoCircolike virus 21 | PClike21 |
| NP_059527.1 | Columbid circovirus | ColCV |
| YP_803549.1 | Finch circovirus | FiCV |
| YP_803546.1 | Gull circovirus | GuCV |
| YP_009110682.1 | Bat circovirus POA/2012/VI | BtPOA/2012/VI |
| YP_009104366.1 | Cyclovirus ZM36a | CyZM36a |
| YP_009091696.1 | Silurus glanis circovirus | SgCV |
| YP_009021891.1 | Mink circovirus | MinkCV |
| YP_007974237.1 | Bat circovirus | BatCV |
| YP_003422530.1 | Porcine circovirus type 1/2a | PCV-1/2a |
| YP_271918.1 | Duck circovirus | DCV |
| NP_573442.1 | Canary circovirus | CaCV |
| NP_150368.1 | Goose circovirus | GoCV |
| YP_009112559.1 | Dromedary stoolassociated circular ssDNA virus | DSaCV |
| YP_009110679.1 | Bat circovirus POA/2012/II | POAII |
| YP_009052458.1 | Feline cyclovirus | FelineCV |
| YP_009021877.1 | Porcine stoolassociated circular virus 5 | PSaCV-5 |
| YP_004152333.1 | Cyclovirus NGchicken15/NGA/2009 | CyNG15 |
| YP_004152331.1 | Cyclovirus bat/USA/2009 | CyBt |
| YP_004152329.1 | Cyclovirus PKgoat11/PAK/2009 | CyPKgoat11 |
| YP_004152327.1 | Cyclovirus PKgoat21/PAK/2009 | CyPKgoat21 |
| YP_764455.1 | Raven circovirus | RaCV |
| YP_009021875.1 | Porcine stoolassociated circular virus 4 | PSaCV-4 |
| YP_009021850.1 | Dragonfly cyclovirus 2 | DaCV2 |
| YP_009021846.1 | Dragonfly cyclovirus 3 | DaCV3 |
| YP_009021845.1 | Dragonfly cyclovirus 4 | DaCV4 |
| YP_009021843.1 | Dragonfly cyclovirus 5 | DaCV5 |
| YP_009051960.1 | Human circovirus VS6600022 | HCVVS6600022 |
| YP_008130363.1 | Human cyclovirus VS5700009 | VS5700009 |
| YP_007697652.1 | Canine circovirus | CanCV |
| YP_007353980.1 | Acartia tonsa copepod circovirus | AtCV |
| YP_006281010.1 | Labidocera aestiva circovirus | LaCV |
| NP_065678.1 | Porcine circovirus1 | PCV-1 |
| NP_047275.1 | Beak and feather disease virus | BFDV |
| YP_009109686.1 | Circoviridae 21 LDMD2013 | LDMD21 |
| YP_009109685.1 | Circoviridae 19 LDMD2013 | LDMD19 |
| YP_009109683.1 | Circoviridae 18 LDMD2013 | LDMD18 |
| YP_009109682.1 | Circoviridae 17 LDMD2013 | LDMD17 |
| YP_009109676.1 | Circoviridae 16 LDMD2013 | LDMD16 |
| YP_009109675.1 | Circoviridae 15 LDMD2013 | LDMD15 |
| YP_009109670.1 | Circoviridae 14 LDMD2013 | LDMD14 |
| YP_009109668.1 | Circoviridae 13 LDMD2013 | LDMD13 |
| YP_009109663.1 | Circoviridae 11 LDMD2013 | LDMD11 |
| YP_009109660.1 | Circoviridae 10 LDMD2013 | LDMD10 |
| YP_009109659.1 | Circoviridae 9 LDMD2013 | LDMD9 |
| YP_009109653.1 | Circoviridae 8 LDMD2013 | LDMD8 |
| YP_009109649.1 | Circoviridae 7 LDMD2013 | LDMD7 |
| YP_009109644.1 | Circoviridae 6 LDMD2013 | LDMD6 |
| YP_009109643.1 | Circoviridae 5 LDMD2013 | LDMD5 |
| YP_009109640.1 | Circoviridae 4 LDMD2013 | LDMD4 |
| YP_009109636.1 | Circoviridae 3 LDMD2013 | LDMD3 |
| YP_009109630.1 | Circoviridae 2 LDMD2013 | LDMD2 |
| YP_009109626.1 | Circoviridae 1 LDMD2013 | LDMD1 |
| YP_009091698.1 | Swan circovirus | SwCV |
| YP_009047065.1 | Cyclovirus VN | CyVN |
| YP_009021893.1 | Dragonfly cyclovirus 1 | DaCV1 |
| YP_009021879.1 | Fur seal faeces associated circular DNA virus | FurSeFaCV |
| YP_009021870.1 | Human cyclovirus | HCyV |
| YP_008828162.1 | Penaeus monodon circovirus VN11 | PmCVN11 |
| YP_009000900.1 | Anguilla anguilla circovirus | AaCV |
| YP_007392931.1 | Florida woods cockroachassociated cyclovirus | FWCaCyV |
| YP_004376332.1 | Barbel circovirus | BaCV |
| YP_610960.1 | Starling circovirus | StarCV |
| NP_937956.1 | Porcine circovirus2 | PCV-2 |
| YP_009021041.1 | Nepavirus | NepaV |
| YP_009051838.1 | MSSI2.225 virus | MSSI2.225 |
| YP_009051835.1 | HCBI9.212 virus | HCB19/212 |
| YP_009051832.1 | HCBI8.215 virus | HCBI8.215 |
| YP_009051837.1 | MSSI2.225 virus | MSSI2.225 |
| YP_009051834.1 | HCBI9.212 virus | HCB19.212 |
| YP_009051831.1 | HCBI8.215 virus | HCBI8.215 |
| YP_003084293.1 | Circoviruslike genome CBA | CB-A |
| YP_003084291.1 | Circoviruslike genome RWE | RW-E |
| YP_003084290.1 | Circoviruslike genome RWD | RW-D |
| YP_003084287.1 | Circoviruslike genome RWC | RW-C |
| YP_003084282.1 | Circoviruslike genome RWA | RW-A |
| YP_003084143.1 | Circoviruslike genome BBCA | BBC-A |
| YP_003084140.1 | Circoviruslike genome SARB | SAR-B |
| YP_009054989.1 | Porcine stoolassociated circular virus 9 | PSaCV-9 |
| YP_009054987.1 | Porcine stoolassociated circular virus 7 | PSaCV-7 |
| YP_009022029.1 | Circolike virusBrazil hs1 | BrazilHS1 |
| YP_003084299.2 | Circoviruslike genome SARA | SAR-A |
| YP_009054993.1 | Porcine stoolassociated circular virus 6 | PSaCV-6 |
| YP_009054991.1 | Porcine stoolassociated circular virus 8 | PSaCV-8 |
| YP_009054985.1 | Porcine stoolassociated circular virus 1 | PSaCV-1 |
| YP_007974230.1 | Porcine stoolassociated circular virus 3 | PSaCV-3 |
| YP_007974228.1 | Porcine stoolassociated circular virus 2 | PSaCV-2 |
| YP_009021856.1 | Dragonflyassociated circular virus 2 | DaCV-2 |
| YP_009021852.1 | Dragonflyassociated circular virus 3 | DaCV-3 |
| YP_009021860.1 | Dragonflyassociated circular virus 1 | DaCV-1a |
| YP_009021245.1 | Dragonfly cyclicusvirus | Cyclo12 |
| YP_009021243.1 | Dragonfly orbiculatusvirus | DOrbiV |
| YP_009021241.1 | Dragonfly circularisvirus | DCV |
| YP_007517186.1 | Gastropod associated circular ssDNA virus | GaCV |
| YP_003084285.1 | Circoviruslike genome RWB | RW-B |
| YP_009116891.1 | Sewageassociated circular DNA virus37 | SaCV-37 |
| YP_009116889.1 | Sewageassociated circular DNA virus36 | SaCV-36 |
| YP_009047130.1 | McMurdo Ice Shelf pondassociated circular DNA virus2 | McMurdo-2 |
| YP_009021862.1 | Dragonflyassociated circular virus 1 | DaCV-1b |
| YP_009021043.1 | Cassava associated cicular DNA virus | Cassava |
| YP_003104796.1 | Sclerotinia sclerotiorum hypovirulence associated DNA virus 1 | SSaCV |
| YP_009117082.1 | Sewageassociated circular DNA virus35 | SaCV-35 |
| YP_009117079.1 | Sewageassociated circular DNA virus34 | SaCV-34 |
| YP_009117078.1 | Sewageassociated circular DNA virus33 | SaCV-33 |
| YP_009117076.1 | Sewageassociated circular DNA virus32 | SaCV-32 |
| YP_009117074.1 | Sewageassociated circular DNA virus31 | SaCV-31 |
| YP_009117070.1 | Sewageassociated circular DNA virus30 | SaCV-30 |
| YP_009117067.1 | Sewageassociated circular DNA virus29 | SaCV-29 |
| YP_009117066.1 | Sewageassociated circular DNA virus28 | SaCV-28 |
| YP_009117061.1 | Sewageassociated circular DNA virus27 | SaCV-27 |
| YP_009117058.1 | Sewageassociated circular DNA virus26 | SaCV-26 |
| YP_009117057.1 | Sewageassociated circular DNA virus25 | SaCV-25 |
| YP_009116913.1 | Sewageassociated circular DNA virus24 | SaCV-24 |
| YP_009116910.1 | Sewageassociated circular DNA virus23 | SaCV-23 |
| YP_009116909.1 | Sewageassociated circular DNA virus22 | SaCV-22 |
| YP_009116906.1 | Sewageassociated circular DNA virus21 | SaCV-21 |
| YP_009116905.1 | Sewageassociated circular DNA virus20 | SaCV-20 |
| YP_009116902.1 | Sewageassociated circular DNA virus19 | SaCV-19 |
| YP_009116898.1 | Sewageassociated circular DNA virus18 | SaCV-18 |
| YP_009116896.1 | Sewageassociated circular DNA virus17 | SaCV-17 |
| YP_009116894.1 | Sewageassociated circular DNA virus16 | SaCV-16 |
| YP_009116892.1 | Sewageassociated circular DNA virus15 | SaCV-15 |
| YP_009047142.1 | McMurdo Ice Shelf pondassociated circular DNA virus7 | McMurdo-7 |
| YP_009047139.1 | McMurdo Ice Shelf pondassociated circular DNA virus6 | McMurdo-6 |
| YP_009047137.1 | McMurdo Ice Shelf pondassociated circular DNA virus5 | McMurdo-5 |
| YP_009047134.1 | McMurdo Ice Shelf pondassociated circular DNA virus4 | McMurdo-4 |
| YP_009047132.1 | McMurdo Ice Shelf pondassociated circular DNA virus3 | McMurdo-3 |
| YP_009047125.1 | McMurdo Ice Shelf pondassociated circular DNA virus1 | McMurdo-1 |
| YP_009001756.1 | Dragonfly larvae associated circular virus10 | DLaCV-10 |
| YP_009001753.1 | Dragonfly larvae associated circular virus9 | DLaCV-9 |
| YP_009001751.1 | Dragonfly larvae associated circular virus8 | DLaCV-8 |
| YP_009001750.1 | Dragonfly larvae associated circular virus7 | DLaCV-7 |
| YP_009001747.1 | Dragonfly larvae associated circular virus6 | DLaCV-6 |
| YP_009001745.1 | Dragonfly larvae associated circular virus5 | DLaCV-5 |
| YP_009001743.1 | Dragonfly larvae associated circular virus4 | DLaCV-4 |
| YP_009001742.1 | Dragonfly larvae associated circular virus3 | DLaCV-3 |
| YP_009001739.1 | Dragonfly larvae associated circular virus2 | DLaCV-2 |
| YP_009001737.1 | Dragonfly larvae associated circular virus1 | DLaCV-1 |
| YP_009021858.1 | Dragonflyassociated circular virus 2 | DaCV-2b |
| YP_009021854.1 | Dragonflyassociated circular virus 3 | DaCV-3a |
| YP_009021890.1 | Cyanoramphus nest associated circular K DNA virus | CNaCVK |
| YP_009021888.1 | Cyanoramphus nest associated circular X DNA virus | CNaCVX |
| YP_007878130.1 | Acheta domesticus volvovirus | AchDVolv |
| YP_003084297.1 | Circoviruslike genome CBB | CB-B |
| YP_006331067.1 | Pig stool associated circular ssDNA virus GER2011 | GER2011 |
| ADB24829.1 | Chimpanzee stool associated circular ssDNA virus | CSaCV1 |
| ADB24823.1 | Chimpanzee stool associated circular ssDNA virus | CSaCV2 |
| ADB24816.1 | Chimpanzee stool associated circular ssDNA virus | CSaCV3 |
| ADB24810.1 | Chimpanzee stool associated circular ssDNA virus | CSaCV4 |
| ADB24805.1 | Chimpanzee stool associated circular ssDNA virus | CSaCV5 |
| ADB24799.1 | Chimpanzee stool associated circular ssDNA virus | CSaCV6 |
| ADB24797.1 | Chimpanzee stool associated circular ssDNA virus | CSaCV7 |
| AEI54346.1 | Picobiliphyte sp. MS5845 nanovirus | MS584-5 |
| AJP36487.1 | AvonHeathcote Estuary associated circular virus 29 | AHEaCV29 |
| AJP36481.1 | AvonHeathcote Estuary associated circular virus 28 | AHEaCV28 |
| AJP36473.1 | AvonHeathcote Estuary associated circular virus 27 | AHEaCV27 |
| AJP36469.1 | AvonHeathcote Estuary associated circular virus 26 | AHEaCV26 |
| AJP36468.1 | AvonHeathcote Estuary associated circular virus 25 | AHEaCV25 |
| AJP36460.1 | AvonHeathcote Estuary associated circular virus 24 | AHEaCV24 |
| AJP36458.1 | AvonHeathcote Estuary associated circular virus 23 | AHEaCV23 |
| AJP36455.1 | AvonHeathcote Estuary associated circular virus 22 | AHEaCV22 |
| AJP36452.1 | AvonHeathcote Estuary associated circular virus 21 | AHEaCV21 |
| AJP36448.1 | AvonHeathcote Estuary associated circular virus 20 | AHEaCV20 |
| AJP36446.1 | AvonHeathcote Estuary associated circular virus 19 | AHEaCV19 |
| AJP36443.1 | AvonHeathcote Estuary associated circular virus 18 | AHEaCV18 |
| AJP36442.1 | AvonHeathcote Estuary associated circular virus 17 | AHEaCV17 |
| AJP36436.1 | AvonHeathcote Estuary associated circular virus 16 | AHEaCV16 |
| AJP36430.1 | AvonHeathcote Estuary associated circular virus 15 | AHEaCV15 |
| AJP36422.1 | AvonHeathcote Estuary associated circular virus 14 | AHEaCV14 |
| AJP36414.1 | AvonHeathcote Estuary associated circular virus 13 | AHEaCV13 |
| AJP36407.1 | AvonHeathcote Estuary associated circular virus 12 | AHEaCV12 |
| AJP36405.1 | AvonHeathcote Estuary associated circular virus 11 | AHEaCV11 |
| AJP36394.1 | AvonHeathcote Estuary associated circular virus 10 | AHEaCV10 |
| AJP36387.1 | AvonHeathcote Estuary associated circular virus 9 | AHEaCV9 |
| AJP36380.1 | AvonHeathcote Estuary associated circular virus 8 | AHEaCV8 |
| AJP36369.1 | AvonHeathcote Estuary associated circular virus 7 | AHEaCV7 |
| AJP36367.1 | AvonHeathcote Estuary associated circular virus 6 | AHEaCV6 |
| AJP36357.1 | AvonHeathcote Estuary associated circular virus 5 | AHEaCV5 |
| AJP36351.1 | AvonHeathcote Estuary associated circular virus 4 | AHEaCV4 |
| AJP36345.1 | AvonHeathcote Estuary associated circular virus 3 | AHEaCV3 |
| AJP36337.1 | AvonHeathcote Estuary associated circular virus 2 | AHEaCV2 |
| AJP36333.1 | AvonHeathcote Estuary associated circular virus 1 | AHEaCV1 |
| AIX11626.1 | Bat circovirus POA/2012/I | POAI |
| AIX11629.1 | Bat circovirus POA/2012/V | POAV |
| AAL00893.1 | Mungbean yellow mosaic India virus(Soybean) | Gemini1 |
| AAM48573.1 | Mungbean yellow mosaic India virus(Cowpea) | Gemini2 |
| AAQ16294.1 | Dolichos yellow mosaic virus | Gemini3 |
| AIL92572.2 | Clerodendron yellow mosaic virus | Gemini4 |
| NP_803150.1 | Mungbean yellow mosaic India virus | Gemini5 |
| AAD20641.1 | Mungbean yellow mosaic India virus | Gemini6 |
| ABF67522.1 | Euphorbia mosaic virus B (Mexico:Jalasco:Pepper:2005) | Gemini7 |
| YP_001210303.1 | Clerodendron yellow mosaic virus | Gemini8 |
| ABP58640.1 | Clerodendron yellow mosaic virus | Gemini9 |
| AAQ16299.1 | Mungbean yellow mosaic virus | Gemini10 |
| ACC95448.1 | Corchorus golden mosaic virus(India:Barrackpore:2008) | Gemini11 |
| AFM38721.1 | Jacquemontia mosaic Yucatan virus | Gemini12 |
| AAP23252.1 | Mungbean yellow mosaic India virus (Nepal) | Gemini13 |
| AAP23244.1 | Mungbean yellow mosaic India virus (Akola) | Gemini14 |
| AEY78477.1 | Croton yellow vein mosaic virus | Gemini15 |
| NP_619769.1 | Milk vetch dwarf virus | Nano1 |
| YP_008992018.1 | Pea necrotic yellow dwarf virus | Nano2 |
| YP_003104737.1 | Faba bean necrotic stunt virus | Nano3 |
| NP_619567.1 | Faba bean necrotic yellows virus | Nano4 |
| AAD11928.1 | Bovine Circovirus | BoCirco |
| ADD62451.1 | Cyclovirus PK5006 | CyPK5006 |
| ADD62453.1 | Human Cyclovirus PK5034 | CyPK5034 |
| ADD62455.1 | Cyclovirus PK5222 | CyPK5222 |
| ADD62457.1 | Cyclovirus PK5510 | PK5510 |
| ADD62461.1 | Cyclovirus Chimp11 | CyChimp11 |
| ADD62463.1 | Cyclovirus Chimp12 | CyChimp12 |
| ADD62471.1 | Human Cyclovirus NG12 | CyNG12 |
| ADD62473.1 | Human Cyclovirus NG14 | CyNG14 |
| ADD62479.1 | Human Cyclovirus TN18 | CyTN18 |
| ADI48251.1 | Bat cyclovirus GF-4c | BatGF4c |
| ADI48253.1 | BatCV TM6C | BatCV TM6C |
| ADU76993.1 | Cyclovirus PKbeef23/PAK/2009 | CyPKBeef |
| AEL87784.1 | Bat circovirus ZS/China/2011 isolate YN-BtCV-1 | YNBtCV1 |
| AEL87786.1 | Bat circovirus ZS/China/2011 isolate YN-BtCV-2 | YNBtCV2 |
| AEL87790.1 | Bat circovirus ZS/China/2011 isolate YN-BtCV-4 | YNBtCV4 |
| AEL87793.1 | Bat circovirus ZS/China/2011 isolate YN-BtCV-5 | YNBtCV5 |
| AEM05795.1 | Rodent stool-associated circular genome virus strain R-15 | RodSCV R 15 |
| AEM05796.1 | Rodent stool-associated circular genome virus strain RodSCV M-89 | RodSCV M 89 |
| AEM05797.1 | Rodent stool-associated circular genome virus strain RodSCV V-69 | RodSCV V 69 |
| AEM05798.1 | Rodent stool-associated circular genome virus strain RodSCV V-76 | RodSCV V 76 |
| AEM05799.1 | Rodent stool-associated circular genome virus strain RodSCV V-77 | RodSCV V 77 |
| AEM05800.1 | Rodent stool-associated circular genome virus strain RodSCV V-87 | RodSCV V 87 |
| AEM05803.1 | Rodent stool-associated circular genome virus strain RodSCV M-44 | RodSCV M 44 |
| AEM05804.1 | Rodent stool-associated circular genome virus strain RodSCV M-45 | RodSCV M 45 |
| AEM05805.1 | Rodent stool-associated circular genome virus strain RodSCV M-13 | RodSCVM13 |
| AEM05806.1 | Rodent stool-associated circular genome virus strain RodSCV V-72 | RodSCV V 72 |
| AEM05807.1 | Rodent stool-associated circular genome virus strain RodSCV V-81 | RodSCV V 81 |
| AEM05808.1 | Rodent stool-associated circular genome virus strain RodSCV V-84 | RodSCV V 84 |
| AEM05809.1 | Rodent stool-associated circular genome virus strain RodSCV V-97 | RodSCV V 97 |
| AEW47007.1 | bovine stool/BK/KOR/2011 isolate CP11-49-3 | BOSVCCP11493 |
| AEW49399.1 | Meles meles circovirus-like virus | MmCV |
| AFH02742.1 | batCV-SC703 | batCV-SC703 |
| AFR68936.1 | Porcine associated stool circular virus Cass | PigSCV |
| AFV77554.1 | Pig stool associated circular ssDNA virus isolate HEN1 | HEN1 |
| AFV77559.1 | Pig stool associated circular ssDNA virus isolate HUB1 | HUB1 |
| AFV77564.1 | Pig stool associated circular ssDNA virus isolate HUB2 | HUB2 |
| AFV77569.1 | Pig stool associated circular ssDNA virus isolate JIANGX1 | JIANGX1 |
| AFV77579.1 | Pig stool associated circular ssDNA virus isolate HUN2 | HUN2 |
| AFV77584.1 | Pig stool associated circular ssDNA virus isolate ANH1 | ANH1 |
| AFV77590.1 | Pig stool associated circular ssDNA virus isolate FUJ1 | FUJ1 |
| AGG39811.1 | Diporeia sp. associated circular virus isolate LM3487 | LM3487 |
| AGG39829.1 | Diporeia sp. associated circular virus isolate LM28925 | LM28925 |
| AGK45262.1 | Porcine stool-associated circular virus 2 isolate f | PoSCV2 |
| AGK45264.1 | Porcine stool-associated circular virus 3 isolate 3L7 | PoSCV33L7 |
| AGK45266.1 | Porcine stool-associated circular virus 3 isolate 4L13 | PoSCV34L13 |
| AGK45268.1 | Porcine stool-associated circular virus 3 isolate 4L5 | PoSCV34L5 |
| AGO61982.1 | Circo-like virus-Brazil hs2 | HS2 |
| AGS47835.1 | Farfantepenaeus duorarum circovirus isolate FL2009 | FdaCV |
| AHF54685.1 | Turkey Stool associated circular virus | TuSCV |
| AIZ46819.1 | Swine Cyclovirus SC CGS77 | CySwine |
| AJD07474.1 | Odanata-associated circular virus-9 | OdasCV-9 |
| AJD07477.1 | Odanata-associated circular virus-1 | OdasCV-1 |
| AJD07478.1 | Odanata-associated circular virus-11 | OdasCV-11 |
| AJD07481.1 | Odanata-associated circular virus-12 | OdasCV-12 |
| AJD07482.1 | Odanata-associated circular virus-13 | OdasCV-13 |
| AJD07485.1 | Odanata-associated circular virus-14 | OdasCV-14 |
| AJD07486.1 | Odanata-associated circular virus-15 | OdasCV-15 |
| AJD07489.1 | Odanata-associated circular virus-2 | OdasCV-2 |
| AJD07490.1 | Odanata-associated circular virus-17 | OdasCV-17 |
| AJD07497.1 | Odanata-associated circular virus-18 | OdasCV-18 |
| AJD07501.1 | Odanata-associated circular virus-19 | OdasCV-19 |
| AJD07502.1 | Odanata-associated circular virus-20 | OdasCV-20 |
| AJD07505.1 | Odanata-associated circular virus-3 | OdasCV-3 |
| AJD07507.1 | Odanata-associated circular virus-4 | OdasCV-4 |
| AJD07508.1 | Odanata-associated circular virus-21 | OdasCV-21 |
| AJD07511.1 | Odanata-associated circular virus-5 | OdasCV-5 |
| AJD07512.1 | Odanata-associated circular virus-16 | OdasCV-16 |
| AJD07514.1 | Odanata-associated circular virus-10 | OdasCV-10 |
| BAP81869.1 | Cyclovirus ZM32 | CyZM32 |
| BAP81871.1 | Cyclovirus ZM01 | CyZM01 |
| BAP81875.1 | Cyclovirus ZM38 | CyZM38 |
| BAP81877.1 | Cyclovirus ZM41 | CyZM41 |
| BAP81879.1 | Cyclovirus ZM50a | CyZM50a |
| BAP81881.1 | Cyclovirus ZM54 | CyZM54 |
| BAP81883.1 | Cyclovirus ZM62 | CyZM62 |
| YP_009091698.1 | Cygnus olor circovirus isolate H51 | CygCirco |
| YP_009121932.1 | Mosquito circovirus strain B19 | MosCirco |
| AEF58757.1 | Mosquito SDWAPI | SDWAPI |
| AEL28813.1 | Bat circovirus bat 00813 | batCV00813 |
| KR186219 | Asterias forbesi associated circular virus | AfasCV |
