# Supplementary material for: Novel circular single-stranded DNA viruses identified in marine invertebrates reveal high sequence diversity and consistent predicted intrinsic disorder patterns within putative structural proteins
Source: Front Microbiol. 2015 Jul 10;6:696. doi: 10.3389/fmicb.2015.00696 (PMC4498126; doi:10.3389/fmicb.2015.00696)
Supplement: Supplementary file 4 [file Table_4.DOCX]

**Supplementary Table S4.** Rep sequences used for phylogenetic reconstruction, their corresponding GenBank accession numbers, a brief descriptive title, and acronyms used for Figure 3.

| Accession | Descriptive Name | Acronym |
| --- | --- | --- |
| JX904075.1 | Uncultured marine virus clone SOG00160 | SOG00160 |
| JX904077.1 | Uncultured marine virus clone SOG00182 | SOG00182 |
| JX904100.1 | Uncultured marine virus clone SOG00665 | SOG00665 |
| JX904106.1 | Uncultured marine virus clone SOG00745 | SOG00745 |
| JX904107.1 | Uncultured marine virus clone SOG00781 | SOG00781 |
| JX904139.1 | Uncultured marine virus clone SOG03994 | SOG03994 |
| JX904144.1 | Uncultured marine virus clone SOG04070 | SOG04070 |
| JX904169.1 | Uncultured marine virus clone SOG04916 | SOG04916 |
| JX904185.1 | Uncultured marine virus clone SOG05268 | SOG05268 |
| JX904191.1 | Uncultured marine virus clone GOM00010 | GOM00010 |
| JX904192.1 | Uncultured marine virus clone GOM00012 | GOM00012 |
| JX904207.1 | Uncultured marine virus clone GOM00189 | GOM00189 |
| JX904221.1 | Uncultured marine virus clone GOM00363 | GOM00363 |
| JX904231.1 | Uncultured marine virus clone GOM00443 | GOM00443 |
| JX904245.1 | Uncultured marine virus clone GOM00546 | GOM00546 |
| JX904250.1 | Uncultured marine virus clone GOM00583 | GOM00583 |
| JX904279.1 | Uncultured marine virus clone GOM00860 | GOM00860 |
| JX904312.1 | Uncultured marine virus clone GOM02856 | GOM02856 |
| JX904333.1 | Uncultured marine virus clone GOM02962 | GOM02962 |
| JX904344.1 | Uncultured marine virus clone GOM03041 | GOM03041 |
| JX904368.1 | Uncultured marine virus clone GOM03161 | GOM03161 |
| JX904377.1 | Uncultured marine virus clone GOM03193 | GOM03193 |
| JX904395.1 | Uncultured marine virus clone SI00006 | SI00006 |
| JX904401.1 | Uncultured marine virus clone SI00063 | SI00063 |
| JX904407.1 | Uncultured marine virus clone SI00078 | SI00078 |
| JX904412.1 | Uncultured marine virus clone SI00094 | SI00094 |
| JX904416.1 | Uncultured marine virus clone SI00142 | SI00142 |
| JX904420.1 | Uncultured marine virus clone SI00197 | SI00197 |
| JX904431.1 | Uncultured marine virus clone SI00373 | SI00373 |
| JX904439.1 | Uncultured marine virus clone SI00441 | SI00441 |
| JX904469.1 | Uncultured marine virus clone SI00793 | SI00793 |
| JX904473.1 | Uncultured marine virus clone SI00850 | SI00850 |
| JX904478.1 | Uncultured marine virus clone SI00898 | SI00898 |
| JX904518.1 | Uncultured marine virus clone SI01664 | SI01664 |
| JX904523.1 | Uncultured marine virus clone SI01813 | SI01813 |
| JX904541.1 | Uncultured marine virus clone SI03513 | SI03513 |
| JX904548.1 | Uncultured marine virus clone SI03654 | SI03654 |
| JX904559.1 | Uncultured marine virus clone SI03701 | SI03701 |
| JX904561.1 | Uncultured marine virus clone SI03705 | SI03705 |
| JX904562.1 | Uncultured marine virus clone SI03717 | SI03717 |
| JX904605.1 | Uncultured marine virus clone SI04276 | SI04276 |
| JX904614.1 | Uncultured marine virus clone SI04298 | SI04298 |
| JX904629.1 | Uncultured marine virus clone SI04364 | SI04364 |
| JX904640.1 | Uncultured marine virus clone SI04410 | SI04410 |
| YP_164517.1 | Muscovy duck circovirus | MuCV |
| NP_877978.1 | Mulard duck circovirus | MulCV |
| YP_009109623.1 | Po-Circo-like virus 51 | PClike51 |
| YP_009109620.1 | Po-Circo-like virus 41 | PClike41 |
| YP_009109615.1 | Po-Circo-like virus 21 | PClike21 |
| NP_059527.1 | Columbid circovirus | CoCV |
| YP_803549.1 | Finch circovirus | FiCV |
| YP_803546.1 | Gull circovirus | GullCV |
| YP_009110682.1 | Bat circovirus POA/2012/VI | BatPOAVI |
| YP_009104366.1 | Cyclovirus ZM36a | CyZM36a |
| YP_009091696.1 | Silurus glanis circovirus | SgCV |
| YP_009021891.1 | Mink circovirus | MinkCV |
| YP_007974237.1 | Bat circovirus | BatCV |
| YP_003422530.1 | Porcine circovirus type 1/2a | PCV1/2a |
| YP_271918.1 | Duck circovirus | DuCV |
| NP_573442.1 | Canary circovirus | CanaCV |
| NP_150368.1 | Goose circovirus | GoCV |
| YP_009112559.1 | Dromedary stool-associated circular ssDNA virus | DSaCV |
| YP_009021877.1 | Porcine stool-associated circular virus 5 | PSaCV-5 |
| YP_004152333.1 | Cyclovirus NGchicken15/NGA/2009 | CyNG15 |
| YP_004152331.1 | Cyclovirus bat/USA/2009 | CyBat09 |
| YP_004152329.1 | Cyclovirus PKgoat11/PAK/2009 | CyPK11 |
| YP_004152327.1 | Cyclovirus PKgoat21/PAK/2009 | CyPK21 |
| YP_764455.1 | Raven circovirus | RavCV |
| YP_009021875.1 | Porcine stool-associated circular virus 4 | PSaCV-4 |
| YP_009021850.1 | Dragonfly cyclovirus 2 | DCy2 |
| YP_009021846.1 | spliced Dragonfly cyclovirus 3 | DCy3 |
| YP_009021845.1 | Dragonfly cyclovirus 4 | DCy4 |
| YP_009021843.1 | Dragonfly cyclovirus 5 | DCy5 |
| YP_009051960.1 | Human circovirus VS6600022 | HaCV022 |
| YP_008130363.1 | Human cyclovirus VS5700009 | HaCV009 |
| YP_007697652.1 | Canine circovirus | CaCV |
| YP_007353980.1 | viral Acartia tonsa copepod circovirus | AtCV |
| YP_006281010.1 | viral Labidocera aestiva circovirus | LaCV |
| NP_065678.1 | Porcine circovirus-1 | PCV1 |
| NP_047275.1 | partial Beak and feather disease virus | BFDV |
| YP_009109686.1 | Circoviridae 21 LDMD-2013 | LDMD21 |
| YP_009109685.1 | Circoviridae 19 LDMD-2013 | LDMD19 |
| YP_009109683.1 | Circoviridae 18 LDMD-2013 | LDMD18 |
| YP_009109682.1 | Circoviridae 17 LDMD-2013 | LDMD17 |
| YP_009109675.1 | Circoviridae 15 LDMD-2013 | LDMD15 |
| YP_009109670.1 | Circoviridae 14 LDMD-2013 | LDMD14 |
| YP_009109668.1 | Circoviridae 13 LDMD-2013 | LDMD13 |
| YP_009109663.1 | Circoviridae 11 LDMD-2013 | LDMD11 |
| YP_009109660.1 | Circoviridae 10 LDMD-2013 | LDMD10 |
| YP_009109659.1 | Circoviridae 9 LDMD-2013 | LDMD9 |
| YP_009109643.1 | Circoviridae 5 LDMD-2013 | LDMD5 |
| YP_009109640.1 | Circoviridae 4 LDMD-2013 | LDMD4 |
| YP_009109630.1 | Circoviridae 2 LDMD-2013 | LDMD2 |
| YP_009109626.1 | Circoviridae 1 LDMD-2013 | LDMD1 |
| YP_009091698.1 | Swan circovirus | SwCV |
| YP_009047065.1 | Cyclovirus VN | CyVN |
| YP_009021893.1 | Dragonfly cyclovirus 1 | DCy1 |
| YP_009021879.1 | Fur seal faeces associated circular DNA virus | FSFaCV |
| YP_009021870.1 | Human cyclovirus | HaCY2 |
| YP_008828162.1 | 1 Penaeus monodon circovirus VN11 | VN11 |
| YP_009000900.1 | Anguilla anguilla circovirus | AaCV |
| YP_004376332.1 | Barbel circovirus | BaCV |
| YP_610960.1 | Starling circovirus | StarCV |
| NP_937956.1 | Porcine circovirus-2 | PoCV2 |
| YP_009021041.1 | Nepavirus | NepaV |
| YP_009051832.1 | HCBI8.215 virus | HCBI8215 |
| YP_003084293.1 | Circovirus-like genome CB-A | CB-A |
| YP_003084291.1 | Circovirus-like genome RW-E | RW-E |
| YP_003084290.1 | Circovirus-like genome RW-D | RW-D |
| YP_003084287.1 | Circovirus-like genome RW-C | RW-C |
| YP_003084282.1 | Circovirus-like genome RW-A | RW-A |
| YP_003084143.1 | Circovirus-like genome BBC-A | BBC-A |
| YP_009054989.1 | Porcine stool-associated circular virus 9 | PSaCV-9 |
| YP_009054987.1 | Porcine stool-associated circular virus 7 | PSaCV-7 |
| YP_009022029.1 | Circo-like virus-Brazil hs1 | BrazilHS1 |
| YP_003084299.2 | Circovirus-like genome SAR-A | SAR-A |
| YP_009054991.1 | Porcine stool-associated circular virus 8 | PSaCV-8 |
| YP_009054985.1 | Porcine stool-associated circular virus 1 | PSaCV-1 |
| YP_007974230.1 | Porcine stool-associated circular virus 3 | PSaCV-3 |
| YP_007974228.1 | Porcine stool-associated circular virus 2 | PSaCV-2 |
| YP_009021856.1 | spliced Dragonfly-associated circular virus 2 | DaCV-2 |
| YP_009021852.1 | spliced Dragonfly-associated circular virus 3 | DaCV-3 |
| YP_009021860.1 | spliced Dragonfly-associated circular virus 1 | DaCV-1 |
| YP_009021245.1 | Dragonfly cyclicusvirus | Dcylic |
| YP_009021243.1 | Dragonfly orbiculatusvirus | DOrbiV |
| YP_009021241.1 | Dragonfly circularisvirus | Dcircular |
| YP_007517186.1 | Gastropod associated circular ssDNA virus | GaCV |
| YP_003084285.1 | Circovirus-like genome RW-B | RW-B |
| YP_009047130.1 | McMurdo Ice Shelf pond-associated circular DNA virus-2 | McMurdo-2 |
| YP_009021862.1 | Dragonfly-associated circular virus 1 | DaCV-1b |
| YP_009021043.1 | Cassava associated cicular DNA virus | Cassava |
| YP_003104796.1 | Sclerotinia sclerotiorum hypovirulence associated DNA virus 1 | SSaCV |
| YP_009117079.1 | Sewage-associated circular DNA virus-34 | SaCV-34 |
| YP_009117078.1 | Sewage-associated circular DNA virus-33 | SaCV-33 |
| YP_009117076.1 | Sewage-associated circular DNA virus-32 | SaCV-32 |
| YP_009117074.1 | Sewage-associated circular DNA virus-31 | SaCV-31 |
| YP_009117070.1 | Sewage-associated circular DNA virus-30 | SaCV-30 |
| YP_009117067.1 | Sewage-associated circular DNA virus-29 | SaCV-29 |
| YP_009117066.1 | Sewage-associated circular DNA virus-28 | SaCV-28 |
| YP_009117061.1 | Sewage-associated circular DNA virus-27 | SaCV-27 |
| YP_009117058.1 | Sewage-associated circular DNA virus-26 | SaCV-26 |
| YP_009117057.1 | Sewage-associated circular DNA virus-25 | SaCV-25 |
| YP_009116910.1 | Sewage-associated circular DNA virus-23 | SaCV-23 |
| YP_009116909.1 | Sewage-associated circular DNA virus-22 | SaCV-22 |
| YP_009116906.1 | Sewage-associated circular DNA virus-21 | SaCV-21 |
| YP_009116905.1 | Sewage-associated circular DNA virus-20 | SaCV-20 |
| YP_009116902.1 | Sewage-associated circular DNA virus-19 | SaCV-19 |
| YP_009116898.1 | Sewage-associated circular DNA virus-18 | SaCV-18 |
| YP_009116896.1 | Sewage-associated circular DNA virus-17 | SaCV-17 |
| YP_009116894.1 | Sewage-associated circular DNA virus-16 | SaCV-16 |
| YP_009047142.1 | McMurdo Ice Shelf pond-associated circular DNA virus-7 | McMurdo7 |
| YP_009047139.1 | McMurdo Ice Shelf pond-associated circular DNA virus-6 | McMurdo-6 |
| YP_009047137.1 | McMurdo Ice Shelf pond-associated circular DNA virus-5 | McMurdo-5 |
| YP_009047134.1 | McMurdo Ice Shelf pond-associated circular DNA virus-4 | McMurdo-4 |
| YP_009047132.1 | McMurdo Ice Shelf pond-associated circular DNA virus-3 | McMurdo-3 |
| YP_009047125.1 | McMurdo Ice Shelf pond-associated circular DNA virus-1 | McMurdo-1 |
| YP_009001756.1 | Dragonfly larvae associated circular virus-10 | DLaCV-10 |
| YP_009001751.1 | Dragonfly larvae associated circular virus-8 | DLaCV-8 |
| YP_009001750.1 | Dragonfly larvae associated circular virus-7 | DLaCV-7 |
| YP_009001747.1 | Dragonfly larvae associated circular virus-6 | DLaCV-6 |
| YP_009001743.1 | Dragonfly larvae associated circular virus-4 | DLaCV-4 |
| YP_009001742.1 | Dragonfly larvae associated circular virus-3 | DLaCV-3 |
| YP_009001739.1 | Dragonfly larvae associated circular virus-2 | DLaCV-2 |
| YP_009001737.1 | Dragonfly larvae associated circular virus-1 | DLaCV-1 |
| YP_009021858.1 | Dragonfly-associated circular virus 2 | DaCV-2b |
| YP_009021890.1 | Cyanoramphus nest associated circular K DNA virus | CNaCVK |
| YP_009021888.1 | Cyanoramphus nest associated circular X DNA virus | CNaCVX |
| ADB24823.1 | Chimpanzee stool associated circular ssDNA virus | CSaCV1 |
| ADB24816.1 | Chimpanzee stool associated circular ssDNA virus | CSaCV2 |
| ADB24810.1 | Chimpanzee stool associated circular ssDNA virus | CSaCV3 |
| ADB24805.1 | Chimpanzee stool associated circular ssDNA virus | CSaCV4 |
| ADB24799.1 | Chimpanzee stool associated circular ssDNA virus | CSaCV5 |
| AEI54346.1 | Picobiliphyte sp. MS584-5 nanovirus | MS584-5 |
| AJP36487.1 | Avon-Heathcote Estuary associated circular virus 29 | AHEaCV29 |
| AJP36473.1 | Avon-Heathcote Estuary associated circular virus 27 | AHEaCV27 |
| AJP36469.1 | Avon-Heathcote Estuary associated circular virus 26 | AHEaCV26 |
| AJP36468.1 | Avon-Heathcote Estuary associated circular virus 25 | AHEaCV25 |
| AJP36460.1 | Avon-Heathcote Estuary associated circular virus 24 | AHEaCV24 |
| AJP36458.1 | Avon-Heathcote Estuary associated circular virus 23 | AHEaCV23 |
| AJP36446.1 | Avon-Heathcote Estuary associated circular virus 19 | AHEaCV19 |
| AJP36443.1 | Avon-Heathcote Estuary associated circular virus 18 | AHEaCV18 |
| AJP36442.1 | Avon-Heathcote Estuary associated circular virus 17 | AHEaCV17 |
| AJP36436.1 | Avon-Heathcote Estuary associated circular virus 16 | AHEaCV16 |
| AJP36430.1 | Avon-Heathcote Estuary associated circular virus 15 | AHEaCV15 |
| AJP36422.1 | Avon-Heathcote Estuary associated circular virus 14 | AHEaCV14 |
| AJP36414.1 | Avon-Heathcote Estuary associated circular virus 13 | AHEaCV13 |
| AJP36407.1 | Avon-Heathcote Estuary associated circular virus 12 | AHEaCV12 |
| AJP36405.1 | Avon-Heathcote Estuary associated circular virus 11 | AHEaCV11 |
| AJP36394.1 | Avon-Heathcote Estuary associated circular virus 10 | AHEaCV10 |
| AJP36387.1 | Avon-Heathcote Estuary associated circular virus 9 | AHEaCV9 |
| AJP36369.1 | Avon-Heathcote Estuary associated circular virus 7 | AHEaCV7 |
| AJP36367.1 | Avon-Heathcote Estuary associated circular virus 6 | AHEaCV6 |
| AJP36357.1 | Avon-Heathcote Estuary associated circular virus 5 | AHEaCV5 |
| AJP36351.1 | Avon-Heathcote Estuary associated circular virus 4 | AHEaCV4 |
| AJP36345.1 | Avon-Heathcote Estuary associated circular virus 3 | AHEaCV3 |
| AJP36337.1 | partial Avon-Heathcote Estuary associated circular virus 2 | AHEaCV2 |
| AJP36333.1 | Avon-Heathcote Estuary associated circular virus 1 | AHEaCV1 |
| AIX11626.1 | Bat circovirus POA/2012/I | POAI |
| AIX11629.1 | Bat circovirus POA/2012/V | POAV |
| AAM48573.1 | Mungbean yellow mosaic India virus-Cowpea | Gemini1 |
| AAQ16294.1 | Dolichos yellow mosaic virus | Gemini2 |
| AIL92572.2 | Clerodendron yellow mosaic virus | Gemini3 |
| NP_803150.1 | Mungbean yellow mosaic India virus | Gemini4 |
| AAD20641.1 | Mungbean yellow mosaic India virus | Gemini5 |
| ABF67522.1 | Euphorbia mosaic virus - B Mexico:Jalasco:Pepper:2005 | Gemini6 |
| YP_001210303.1 | Clerodendron yellow mosaic virus | Gemini7 |
| ABP58640.1 | Clerodendron yellow mosaic virus | Gemini8 |
| AAQ16299.1 | Mungbean yellow mosaic virus | Gemini9 |
| ACC95448.1 | Corchorus golden mosaic virus-India:Barrackpore:2008 | Gemini10 |
| AFM38721.1 | Jacquemontia mosaic Yucatan virus | Gemini11 |
| AAP23252.1 | Mungbean yellow mosaic India virus - Nepal | Gemini12 |
| AAP23244.1 | Mungbean yellow mosaic India virus - Akola | Gemini13 |
| AEY78477.1 | Croton yellow vein mosaic virus | Gemini14 |
| NP_619769.1 | Milk vetch dwarf virus | Nano1 |
| YP_008992018.1 | Pea necrotic yellow dwarf virus | Nano2 |
| YP_003104737.1 | Faba bean necrotic stunt virus | Nano3 |
| NP_619567.1 | Faba bean necrotic yellows virus | Nano4 |
| AAD11928.1 | and coat bovine circovirus | BoCirco |
| ADD62451.1 | Cyclovirus PK5006 | CyPK5006 |
| ADD62453.1 | Cyclovirus PK5034 | CyPK5034 |
| ADD62455.1 | Cyclovirus PK5222 | CyPK5222 |
| ADD62457.1 | Cyclovirus PK5510 | PK5510 |
| ADD62461.1 | Cyclovirus Chimp11 | CyChimp11 |
| ADD62463.1 | Cyclovirus Chimp12 | CyChimp12 |
| ADD62471.1 | Cyclovirus NG12 | CyNG12 |
| ADD62473.1 | Cyclovirus NG14 | CyNG14 |
| ADD62479.1 | Cyclovirus TN18 | CyTN18 |
| ADI48251.1 | Bat cyclovirus GF-4c | BatGF4c |
| ADI48253.1 | Circoviridae TM-6c | BatCVTM6C |
| ADU76993.1 | Cyclovirus PKbeef23/PAK/2009 | CyPKBeef |
| AEL87786.1 | Bat circovirus ZS/China/2011 | BatZS1 |
| AEL87790.1 | Bat circovirus ZS/China/2011 | BatZS2 |
| AEM05795.1 | Rodent stool-associated circular genome virus | RodSCV1 |
| AEM05796.1 | Rodent stool-associated circular genome virus | RodSCV2 |
| AEM05797.1 | Rodent stool-associated circular genome virus | RodSCV3 |
| AEM05798.1 | Rodent stool-associated circular genome virus | RodSCV4 |
| AEM05799.1 | Rodent stool-associated circular genome virus | RodSCV5 |
| AEM05800.1 | Rodent stool-associated circular genome virus | RodSCV6 |
| AEM05805.1 | Rodent stool-associated circular genome virus | RodSCV7 |
| AEM05806.1 | Rodent stool-associated circular genome virus | RodSCV8 |
| AEM05807.1 | Rodent stool-associated circular genome virus | RodSCV9 |
| AEM05808.1 | Rodent stool-associated circular genome virus | RodSCV10 |
| AEM05809.1 | Rodent stool-associated circular genome virus | RodSCV11 |
| AEW47007.1 | Circoviridae bovine stool/BK/KOR/2011 | BKKor |
| AEW49399.1 | Meles meles circovirus-like virus | MmCV |
| AFH02742.1 | Circoviridae batCV-SC703 | btCVSC703 |
| AFR68936.1 | Porcine associated stool circular virus | PigSCV |
| AGG39811.1 | Diporeia sp. associated circular virus | LM3487 |
| AGG39829.1 | Diporeia sp. associated circular virus | LM28925 |
| AGK45262.1 | Porcine stool-associated circular virus 2 | PoSCV2 |
| AGK45264.1 | Porcine stool-associated circular virus 3 | PoSCV33L7 |
| AGK45266.1 | Porcine stool-associated circular virus 3 | PoSC34L13 |
| AGK45268.1 | Porcine stool-associated circular virus 3 | PoSCV34L5 |
| AGO61982.1 | Circo-like virus-Brazil hs2 | HS2 |
| AGS47835.1 | Farfantepenaeus duorarum circovirus | FdaCV |
| AHF54685.1 | Turkey stool associated circular ssDNA virus | TuSCV |
| AIZ46819.1 | Swine cyclovirus | CySwine |
| AJD07474.1 | Odonata-associated circular virus-9 | OdasCV-9 |
| AJD07477.1 | Odonata-associated circular virus-1 | OdasCV-1 |
| AJD07478.1 | Odonata-associated circular virus-11 | OdasCV-11 |
| AJD07481.1 | Odonata-associated circular virus-12 | OdasCV-12 |
| AJD07482.1 | Odonata-associated circular virus-13 | OdasCV-13 |
| AJD07485.1 | Odonata-associated circular virus-14 | OdasCV-14 |
| AJD07486.1 | Odonata-associated circular virus-15 | OdasCV-15 |
| AJD07489.1 | Odonata-associated circular virus-2 | OdasCV-2 |
| AJD07490.1 | Odonata-associated circular virus-17 | OdasCV-17 |
| AJD07497.1 | Odonata-associated circular virus-18 | OdasCV-18 |
| AJD07501.1 | Odonata-associated circular virus-19 | OdasCV-19 |
| AJD07502.1 | Odonata-associated circular virus-20 | OdasCV-20 |
| AJD07505.1 | Odonata-associated circular virus-3 | OdasCV-3 |
| AJD07507.1 | Odonata-associated circular virus-4 | OdasCV-4 |
| AJD07511.1 | Odonata-associated circular virus-5 | OdasCV-5 |
| AJD07512.1 | Odonata-associated circular virus-16 | OdasCV-16 |
| AJD07514.1 | Odonata-associated circular virus-10 | OdasCV-10 |
| BAP81869.1 | Cyclovirus ZM32 | CyZM32 |
| BAP81871.1 | Cyclovirus ZM01 | CyZM01 |
| BAP81875.1 | Cyclovirus ZM38 | CyZM38 |
| BAP81877.1 | Cyclovirus ZM41 | CyZM41 |
| BAP81879.1 | Cyclovirus ZM50a | CyZM50a |
| BAP81881.1 | Cyclovirus ZM54 | CyZM54 |
| BAP81883.1 | Cyclovirus ZM62 | CyZM62 |
| YP_009121932.1 | Mosquito circovirus B-19 | MosB19CV |
| AEL28813.1 | Bat circovirus batCV00813 | Bat00813 |
| AEF58757.1 | Mosquito circovirus SDWAPI | SDWAPI |
| KR186219 | Asterias forbesi associated circular virus | AfasCV |
