# Supplementary material for: Novel circular single-stranded DNA viruses identified in marine invertebrates reveal high sequence diversity and consistent predicted intrinsic disorder patterns within putative structural proteins
Source: Front Microbiol. 2015 Jul 10;6:696. doi: 10.3389/fmicb.2015.00696 (PMC4498126; doi:10.3389/fmicb.2015.00696)
Supplement: Supplementary file 5 [file Table_5.DOCX]

**Supplementary Table S5** Results of DisProt VL3 IDP predictions for each Cap and non-Rep encoding major ORF analyzed denoted by either Type A, Type B, Neither, or none. Asterisks (*) were used to show major ORFs detected as capsids based on the IDP analysis within genomes that were either not annotated as either having a 2^nd^ major ORF or did not have BLAST hits to annotated Caps. Genomes that did not have a 2^nd^ major ORF were labeled as none for the Capsid IDP Type. Designations have been given for use in this study. Env corresponds to metagenomic CRESS-DNA viruses.

| Descriptive Title | Capsid/Genome Accession | Capsid IDP Type | Designation |
| --- | --- | --- | --- |
| Bat circovirus ZS/China/2011 isolate YN-BtCV-1 | AEL87785.1 | Neither | Cyclo |
| Dragonfly cyclovirus 2 | YP_009021849.1 | Neither | Cyclo |
| Avon-Heathcote Estuary associated circular virus 12 | YP_009126902.1 | Neither | Env |
| Avon-Heathcote Estuary associated circular virus 28 | YP_009126886.1 | Neither | Env |
| Avon-Heathcote Estuary associated circular virus 29 | YP_009126888.1 | Neither | Env |
| Avon-Heathcote Estuary associated circular virus 7 | YP_009126893.1 | Neither | Env |
| Barbel circovirus | YP_004376333.1 | Neither | Env |
| Chimpanzee stool associated circular ssDNA virus | ADB24824.1 | Neither | Env |
| Chimpanzee stool associated circular ssDNA virus | ADB24817.1 | Neither | Env |
| Chimpanzee stool associated circular ssDNA virus | ADB24811.1 | Neither | Env |
| Chimpanzee stool associated circular ssDNA virus | ADB24806.1 | Neither | Env |
| Chimpanzee stool associated circular ssDNA virus | ADB24800.1 | Neither | Env |
| Chimpanzee stool associated circular ssDNA virus | ADB24798.1 | Neither | Env |
| Circoviridae 21 LDMD-2013 | YP_009109687.1 | Neither | Env |
| Dragonfly larvae associated circular virus-8 | YP_009001752.1 | Neither | Env |
| Farfantepenaeus duorarum circovirus isolate FL2009 | AGS47836.1 | Neither | Env |
| Odanata-associated circular virus-3 | AJD07504.1 | Neither | Env |
| Pig stool associated circular ssDNA virus isolate FUJ1 | AFV77589.1 | Neither | Env |
| Porcine stool-associated circular virus 3 | YP_007974229.1 | Neither | Env |
| Porcine stool-associated circular virus 3 isolate 3L7 | AGK45263.1 | Neither | Env |
| Porcine stool-associated circular virus 3 isolate 4L13 | AGK45265.1 | Neither | Env |
| Porcine stool-associated circular virus 3 isolate 4L5 | AGK45267.1 | Neither | Env |
| Porcine stool-associated circular virus 7 | YP_009054986.1 | Neither | Env |
| Rodent stool-associated circular genome virus strain RodSCV_V-77 | JF755405.1 | Neither | Env |
| Rodent stool-associated circular genome virus strain RodSCV_V-87 | JF755406.1 | Neither | Env |
| Sewage-associated circular DNA virus-22 | YP_009116908.1 | Neither | Env |
| Odanata-associated circular virus-21 | AJD07509.1 | Neither | Env |
| Odanata-associated circular virus-5 | AJD07510.1 | Neither | Env |
| Pig stool associated circular ssDNA virus GER2011 | YP_006331068.1 | Neither | Env |
| Pig stool associated circular ssDNA virus isolate ANH1 | AFV77585.1 | Neither | Env |
| Pig stool associated circular ssDNA virus isolate HEN1 | AFV77555.1 | Neither | Env |
| Pig stool associated circular ssDNA virus isolate HUB1 | AFV77560.1 | Neither | Env |
| Pig stool associated circular ssDNA virus isolate HUB2 | AFV77565.1 | Neither | Env |
| Pig stool associated circular ssDNA virus isolate HUN2 | AFV77580.1 | Neither | Env |
| Pig stool associated circular ssDNA virus isolate JIANGX1 | AFV77570.1 | Neither | Env |
| Porcine associated stool circular virus Cass | AFR68937.1 | Neither | Env |
| Porcine stool-associated circular virus 1 | YP_009054984.1 | Neither | Env |
| Porcine stool-associated circular virus 2 | YP_007974227.1 | Neither | Env |
| Porcine stool-associated circular virus 2 isolate f | AGK45261.1 | Neither | Env |
| Porcine stool-associated circular virus 8 | YP_009054990.1 | Neither | Env |
| Porcine stool-associated circular virus 9 | YP_009054988.1 | Neither | Env |
| Turkey Stool associated circular virus | AHF54686.1 | Neither | Env |
| Uncultured marine virus clone SI00898 | JX904478.1 | Neither | Env |
| Avon-Heathcote Estuary associated circular virus 14 | YP_009126914.1 | Neither | Env |
| Avon-Heathcote Estuary associated circular virus 20 | YP_009126926.1 | Neither | Env |
| Nepavirus | YP_009021040.1 | Neither | Env |
| Sewage-associated circular DNA virus-27 | YP_009117060.1 | Neither | Env |
| Acartia tonsa copepod circovirus | YP_007353981.1 | Neither | Env |
| Circovirus-like genome SAR-A | YP_003084298.2 | Neither | Env |
| Rodent stool-associated circular genome virus strain RodSCV_M-45 | JF755409.1 | Neither | Env |
| *P. pacifica* Coral associated circular virus (I0345) | Pending | Neither | This Study |
| Sporobolus striate mosaic virus 1 | YP_006666526.1 | Neither | Gemini |
| Uncultured marine virus clone GOM00363 | JX904221.1 | none | Env |
| Uncultured marine virus clone GOM00546 | JX904245.1 | none | Env |
| Uncultured marine virus clone GOM00583 | JX904250.1 | none | Env |
| Uncultured marine virus clone GOM01500 | JX904301.1 | none | Env |
| Uncultured marine virus clone GOM02962 | JX904333.1 | none | Env |
| Uncultured marine virus clone GOM03041 | JX904344.1 | none | Env |
| Uncultured marine virus clone GOM03098 | JX904346.1 | none | Env |
| Uncultured marine virus clone GOM03116 | JX904359.1 | none | Env |
| Uncultured marine virus clone GOM03161 | JX904368.1 | none | Env |
| Uncultured marine virus clone GOM03175 | JX904373.1 | none | Env |
| Uncultured marine virus clone GOM03193 | JX904377.1 | none | Env |
| Uncultured marine virus clone GOM03194 | JX904378.1 | none | Env |
| Uncultured marine virus clone SI00006 | JX904395.1 | none | Env |
| Uncultured marine virus clone SI00063 | JX904401.1 | none | Env |
| Uncultured marine virus clone SI00067 | JX904403.1 | none | Env |
| Uncultured marine virus clone SI00102 | JX904415.1 | none | Env |
| Uncultured marine virus clone SI00197 | JX904420.1 | none | Env |
| Uncultured marine virus clone SI00373 | JX904431.1 | none | Env |
| Uncultured marine virus clone SI00441 | JX904439.1 | none | Env |
| Uncultured marine virus clone SI00841 | JX904472.1 | none | Env |
| Uncultured marine virus clone SI00850 | JX904473.1 | none | Env |
| Uncultured marine virus clone SI01524 | JX904511.1 | none | Env |
| Uncultured marine virus clone SI01664 | JX904518.1 | none | Env |
| Uncultured marine virus clone SI01813 | JX904523.1 | none | Env |
| Uncultured marine virus clone SI03691 | JX904555.1 | none | Env |
| Uncultured marine virus clone SI03701 | JX904559.1 | none | Env |
| Uncultured marine virus clone SI03717 | JX904562.1 | none | Env |
| Uncultured marine virus clone SI03747 | JX904566.1 | none | Env |
| Uncultured marine virus clone SI03890 | JX904575.1 | none | Env |
| Uncultured marine virus clone SI03931 | JX904581.1 | none | Env |
| Uncultured marine virus clone SI04276 | JX904605.1 | none | Env |
| Uncultured marine virus clone SI04364 | JX904629.1 | none | Env |
| Uncultured marine virus clone SI04406 | JX904639.1 | none | Env |
| Uncultured marine virus clone SI04458 | JX904647.1 | none | Env |
| Uncultured marine virus clone SI04504 | JX904655.1 | none | Env |
| Uncultured marine virus clone SI04666 | JX904674.1 | none | Env |
| Uncultured marine virus clone SOG00160 | JX904075.1 | none | Env |
| Uncultured marine virus clone SOG00182 | JX904077.1 | none | Env |
| Uncultured marine virus clone SOG00568 | JX904092.1 | none | Env |
| Uncultured marine virus clone SOG00593 | JX904094.1 | none | Env |
| Uncultured marine virus clone SOG00665 | JX904100.1 | none | Env |
| Uncultured marine virus clone SOG00690 | JX904101.1 | none | Env |
| Uncultured marine virus clone SOG00745 | JX904106.1 | none | Env |
| Uncultured marine virus clone SOG00781 | JX904107.1 | none | Env |
| Uncultured marine virus clone SOG00824 | JX904108.1 | none | Env |
| Uncultured marine virus clone SOG00852 | JX904109.1 | none | Env |
| Uncultured marine virus clone SOG03994 | JX904139.1 | none | Env |
| Uncultured marine virus clone SOG04311 | JX904151.1 | none | Env |
| Uncultured marine virus clone SOG04508 | JX904155.1 | none | Env |
| Uncultured marine virus clone SOG04916 | JX904169.1 | none | Env |
| Uncultured marine virus clone SOG05268 | JX904185.1 | none | Env |
| Chimpanzee stool associated circular ssDNA virus | ADB24797.1 | none | Env |
| Circoviridae 19 LDMD-2013 | YP_009109685.1 | none | Env |
| Circoviridae 7 LDMD-2013 | YP_009109649.1 | none | Env |
| Circoviridae 9 LDMD-2013 | YP_009109659.1 | none | Env |
| Picobiliphyte sp. MS584-5 nanovirus | AEI54346.1 | none | Env |
| Po-Circo-like virus 41 | YP_009109620.1 | none | Env |
| Po-Circo-like virus 51 | YP_009109623.1 | none | Env |
| Rodent stool-associated circular genome virus strain R-15 | JF755401.1 | none | Env |
| Rodent stool-associated circular genome virus strain RodSCV_M-13 | JF755410.1 | none | Env |
| Rodent stool-associated circular genome virus strain RodSCV_M-44 | JF755408.1 | none | Env |
| Rodent stool-associated circular genome virus strain RodSCV_M-89 | JF755402.1 | none | Env |
| Rodent stool-associated circular genome virus strain RodSCV_V-76 | JF755404.1 | none | Env |
| Sewage-associated circular DNA virus-31 | YP_009117074.1 | none | Env |
| Canary circovirus | NP_573443.1 | Type A | Circo |
| Columbid circovirus | NP_059530.1 | Type A | Circo |
| Dragonfly cyclovirus 1 | YP_009021894.1 | Type A | Circo |
| Duck circovirus | YP_271921.1 | Type A | Circo |
| Goose circovirus | NP_150370.1 | Type A | Circo |
| Gull circovirus | YP_803548.1 | Type A | Circo |
| Mulard duck circovirus | NP_877979.1 | Type A | Circo |
| Muscovy duck circovirus | YP_164519.1 | Type A | Circo |
| Porcine circovirus type 1/2a | YP_003422531.1 | Type A | Circo |
| Porcine circovirus-1 | NP_065679.1 | Type A | Circo |
| Porcine circovirus-2 | NP_937957.1 | Type A | Circo |
| Raven circovirus | YP_764456.1 | Type A | Circo |
| Starling circovirus | YP_610962.1 | Type A | Circo |
| Swan circovirus | YP_009091699.1 | Type A | Circo |
| Dragonfly cyclovirus 3 | YP_009021848.1 | Type A | Cyclo |
| Bat circovirus ZS/China/2011 isolate YN-BtCV-2 | AEL87787.1 | Type A | Cyclo |
| Bat circovirus ZS/China/2011 isolate YN-BtCV-4 | AEL87791.1 | Type A | Cyclo |
| Bat circovirus ZS/China/2011 isolate YN-BtCV-5 | AEL87793.1 | Type A | Cyclo |
| Bat cyclovirus GF-4c | ADI48252.1 | Type A | Cyclo |
| Cyclovirus bat/USA/2009 | YP_004152332.1 | Type A | Cyclo |
| Cyclovirus Chimp11 | ADD62462.1 | Type A | Cyclo |
| Cyclovirus Chimp12 | ADD62464.1 | Type A | Cyclo |
| Cyclovirus NGchicken15/NGA/2009 | YP_004152334.1 | Type A | Cyclo |
| Cyclovirus PK5006 | ADD62452.1 | Type A | Cyclo |
| Cyclovirus PK5222 | ADD62456.1 | Type A | Cyclo |
| Cyclovirus PK5510 | ADD62458.1 | Type A | Cyclo |
| Cyclovirus PKbeef23/PAK/2009 | ADU76994.1 | Type A | Cyclo |
| Cyclovirus PKgoat11/PAK/2009 | YP_004152330.1 | Type A | Cyclo |
| Cyclovirus PKgoat21/PAK/2009 | YP_004152328.1 | Type A | Cyclo |
| Cyclovirus VN | YP_009047066.1 | Type A | Cyclo |
| Cyclovirus ZM01 | BAP81870.1 | Type A | Cyclo |
| Cyclovirus ZM32 | BAP81868.1 | Type A | Cyclo |
| Cyclovirus ZM36a | YP_009104365.1 | Type A | Cyclo |
| Cyclovirus ZM38 | BAP81874.1 | Type A | Cyclo |
| Cyclovirus ZM41 | BAP81876.1 | Type A | Cyclo |
| Cyclovirus ZM50a | BAP81878.1 | Type A | Cyclo |
| Cyclovirus ZM54 | BAP81880.1 | Type A | Cyclo |
| Cyclovirus ZM62 | BAP81882.1 | Type A | Cyclo |
| Dragonfly cyclovirus 3 | YP_009021848.1 | Type A | Cyclo |
| Dragonfly cyclovirus 4 | YP_009021844.1 | Type A | Cyclo |
| Dragonfly cyclovirus 5 | YP_009021842.1 | Type A | Cyclo |
| Feline cyclovirus | YP_009052459.1 | Type A | Cyclo |
| Florida woods cockroach-associated cyclovirus | YP_007392930.1 | Type A | Cyclo |
| Human cyclovirus | YP_009021871.1 | Type A | Cyclo |
| Human Cyclovirus NG12 | ADD62472.1 | Type A | Cyclo |
| Human Cyclovirus NG14 | ADD62474.1 | Type A | Cyclo |
| Human Cyclovirus PK5034 | ADD62454.1 | Type A | Cyclo |
| Human Cyclovirus TN18 | ADD62480.1 | Type A | Cyclo |
| Human cyclovirus VS5700009 | YP_008130364.1 | Type A | Cyclo |
| Uncultured marine virus clone GOM00010 | JX904191.1 | Type A | Env |
| Uncultured marine virus clone GOM00012 | JX904192.1 | Type A | Env |
| Uncultured marine virus clone GOM00857 | JX904278.1 | Type A | Env |
| Uncultured marine virus clone SI00142 | JX904416.1 | Type A | Env |
| Uncultured marine virus clone SI00349 | JX904427.1 | Type A | Env |
| Uncultured marine virus clone SI00793 | JX904469.1 | Type A | Env |
| Uncultured marine virus clone SI03654 | JX904548.1 | Type A | Env |
| Uncultured marine virus clone SI04410 | JX904640.1 | Type A | Env |
| Uncultured marine virus clone SOG00164 | JX904076.1 | Type A | Env |
| Uncultured marine virus clone SOG00640 | JX904098.1 | Type A | Env |
| Uncultured marine virus clone SOG00662 | JX904099.1 | Type A | Env |
| Uncultured marine virus clone SOG01527 | JX904121.1 | Type A | Env |
| Uncultured marine virus clone SOG03823 | JX904134.1 | Type A | Env |
| Uncultured marine virus clone SOG04070 | JX904144.1 | Type A | Env |
| Uncultured marine virus clone SOG04928 | JX904172.1 | Type A | Env |
| Acheta domesticus volvovirus | AGE84323.1 | Type A | Env |
| Anguilla anguilla circovirus | YP_009000901.1 | Type A | Env |
| Asterias forbesi associated circular virus | KR186219 | Type A | Env |
| Avon-Heathcote Estuary associated circular virus 1 | YP_009126933.1 | Type A | Env |
| Avon-Heathcote Estuary associated circular virus 10 | YP_009126899.1 | Type A | Env |
| Avon-Heathcote Estuary associated circular virus 11 | YP_009126900.1 | Type A | Env |
| Avon-Heathcote Estuary associated circular virus 16 | YP_009126918.1 | Type A | Env |
| Avon-Heathcote Estuary associated circular virus 17 | YP_009126923.1 | Type A | Env |
| Avon-Heathcote Estuary associated circular virus 18 | YP_009126921.1 | Type A | Env |
| Avon-Heathcote Estuary associated circular virus 19 | YP_009126924.1 | Type A | Env |
| Avon-Heathcote Estuary associated circular virus 2 | YP_009126939.1 | Type A | Env |
| Avon-Heathcote Estuary associated circular virus 21 | YP_009126928.1 | Type A | Env |
| Avon-Heathcote Estuary associated circular virus 22 | YP_009126931.1 | Type A | Env |
| Avon-Heathcote Estuary associated circular virus 23 | YP_009126934.1 | Type A | Env |
| Avon-Heathcote Estuary associated circular virus 24 | YP_009126937.1 | Type A | Env |
| Avon-Heathcote Estuary associated circular virus 25 | YP_009126940.1 | Type A | Env |
| Avon-Heathcote Estuary associated circular virus 27 | YP_009126880.1 | Type A | Env |
| Avon-Heathcote Estuary associated circular virus 3 | YP_009126876.1 | Type A | Env |
| Avon-Heathcote Estuary associated circular virus 6 | YP_009126891.1 | Type A | Env |
| Avon-Heathcote Estuary associated circular virus 8 | YP_009126894.1 | Type A | Env |
| Avon-Heathcote Estuary associated circular virus 9 | YP_009126897.1 | Type A | Env |
| Bat circovirus | YP_007974238.1 | Type A | Env |
| Bat circovirus POA/2012/II | YP_009110680.1 | Type A | Env |
| Bat circovirus POA/2012/VI | YP_009110681.1 | Type A | Env |
| BatCV TM6C | ADI48254.1 | Type A | Env |
| batCV-SC703 | AFH02743.1 | Type A | Env |
| Bovine Circovirus | AAD11929.1 | Type A | Env |
| Canine circovirus | YP_007697653.1 | Type A | Env |
| Cassava associated cicular DNA virus | YP_009021042.1 | Type A | Env |
| Circo-like virus-Brazil hs2 | AGO61981.1 | Type A | Env |
| Circoviridae 1 LDMD-2013 | YP_009109629.1 | Type A | Env |
| Circoviridae 15 LDMD-2013 | YP_009109673.1 | Type A | Env |
| Circoviridae 17 LDMD-2013 | YP_009109681.1 | Type A | Env |
| Circoviridae 2 LDMD-2013 | YP_009109631.1 | Type A | Env |
| Circoviridae 4 LDMD-2013 | YP_009109639.1 | Type A | Env |
| Circoviridae 8 LDMD-2013 | YP_009109656.1 | Type A | Env |
| Circovirus-like genome RW-A | YP_003084283.1 | Type A | Env |
| Circovirus-like genome RW-B | YP_003084286.1 | Type A | Env |
| Circovirus-like genome RW-C | YP_003084288.1 | Type A | Env |
| Circovirus-like genome RW-E | YP_003084292.1 | Type A | Env |
| Cyanoramphus nest associated circular K DNA virus | YP_009021889.1 | Type A | Env |
| Cyanoramphus nest associated circular X DNA virus | YP_009021887.1 | Type A | Env |
| Cygnus olor circovirus isolate H51 | YP_009091699.1 | Type A | Env |
| Diporeia sp. associated circular virus isolate LM28925 | AGG39828.1 | Type A | Env |
| Dragonfly cyclicusvirus | YP_009021246.1 | Type A | Env |
| Dragonfly larvae associated circular virus-3 | YP_009001741.1 | Type A | Env |
| Dragonfly larvae associated circular virus-5 | YP_009001746.1 | Type A | Env |
| Dragonfly larvae associated circular virus-6 | YP_009001748.1 | Type A | Env |
| Dragonfly larvae associated circular virus-9 | YP_009001754.1 | Type A | Env |
| Dragonfly orbiculatusvirus | YP_009021244.1 | Type A | Env |
| Dragonfly-associated circular virus 2 | YP_009021855.1 | Type A | Env |
| Dragonfly-associated circular virus 3 | YP_009021851.1 | Type A | Env |
| Dromedary stool-associated circular ssDNA virus | YP_009112561.1 | Type A | Env |
| Gastropod associated circular ssDNA virus | YP_007517185.1 | Type A | Env |
| HCBI8.215 virus | YP_009051830.1 | Type A | Env |
| HCBI9.212 virus | YP_009051833.1 | Type A | Env |
| Human circovirus VS6600022 | YP_009051962.1 | Type A | Env |
| Labidocera aestiva circovirus | YP_006281011.2 | Type A | Env |
| McMurdo Ice Shelf pond-associated circular DNA virus-2 | YP_009047129.1 | Type A | Env |
| McMurdo Ice Shelf pond-associated circular DNA virus-3 | YP_009047133.1 | Type A | Env |
| McMurdo Ice Shelf pond-associated circular DNA virus-5 | YP_009047138.1 | Type A | Env |
| McMurdo Ice Shelf pond-associated circular DNA virus-6 | YP_009047140.1 | Type A | Env |
| McMurdo Ice Shelf pond-associated circular DNA virus-7 | YP_009047143.1 | Type A | Env |
| Meles meles circovirus-like virus | AEW49400.1 | Type A | Env |
| Mink circovirus | YP_009021892.1 | Type A | Env |
| Mosquito circovirus strain B19 | YP_009121933.1 | Type A | Env |
| MSSI2.225 virus | YP_009051836.1 | Type A | Env |
| Odanata-associated circular virus-1 | AJD07476.1 | Type A | Env |
| Odanata-associated circular virus-10 | AJD07515.1 | Type A | Env |
| Odanata-associated circular virus-11 | AJD07479.1 | Type A | Env |
| Odanata-associated circular virus-13 | AJD07483.1 | Type A | Env |
| Odanata-associated circular virus-14 | AJD07484.1 | Type A | Env |
| Odanata-associated circular virus-16 | AJD07513.1 | Type A | Env |
| Odanata-associated circular virus-18 | AJD07492.1 | Type A | Env |
| Odanata-associated circular virus-19 | AJD07500.1 | Type A | Env |
| Odanata-associated circular virus-2 | AJD07488.1 | Type A | Env |
| Odanata-associated circular virus-4 | AJD07506.1 | Type A | Env |
| Odanata-associated circular virus-9 | AJD07475.1 | Type A | Env |
| Penaeus monodon circovirus VN11 | YP_008828163.1 | Type A | Env |
| Porcine stool-associated circular virus 5 | YP_009021876.1 | Type A | Env |
| Porcine stool-associated circular virus 6 | YP_009054992.1 | Type A | Env |
| Sclerotinia sclerotiorum hypovirulence associated DNA virus 1 | AJD07458.1 | Type A | Env |
| Sewage-associated circular DNA virus-15 | YP_009116893.1 | Type A | Env |
| Sewage-associated circular DNA virus-16 | YP_009116895.1 | Type A | Env |
| Sewage-associated circular DNA virus-17 | YP_009116897.1 | Type A | Env |
| Sewage-associated circular DNA virus-18 | YP_009116899.1 | Type A | Env |
| Sewage-associated circular DNA virus-20 | YP_009116904.1 | Type A | Env |
| Sewage-associated circular DNA virus-24 | YP_009116912.1 | Type A | Env |
| Sewage-associated circular DNA virus-25 | YP_009117055.1 | Type A | Env |
| Sewage-associated circular DNA virus-26 | YP_009117059.1 | Type A | Env |
| Sewage-associated circular DNA virus-28 | YP_009117065.1 | Type A | Env |
| Sewage-associated circular DNA virus-29 | YP_009117069.1 | Type A | Env |
| Sewage-associated circular DNA virus-30 | YP_009117071.1 | Type A | Env |
| Sewage-associated circular DNA virus-32 | YP_009117075.1 | Type A | Env |
| Sewage-associated circular DNA virus-33 | YP_009117077.1 | Type A | Env |
| Sewage-associated circular DNA virus-34 | YP_009117080.1 | Type A | Env |
| Sewage-associated circular DNA virus-35 | YP_009117081.1 | Type A | Env |
| Sewage-associated circular DNA virus-36 | YP_009116888.1 | Type A | Env |
| Sewage-associated circular DNA virus-37 | YP_009116890.1 | Type A | Env |
| Silurus glanis circovirus | YP_009091697.1 | Type A | Env |
| Swine Cyclovirus SC_CGS77 | AIZ46820.1 | Type A | Env |
| Avon-Heathcote Estuary associated circular virus 15 | YP_009126917.1 | Type A | Env |
| Avon-Heathcote Estuary associated circular virus 5 | YP_009126885.1 | Type A | Env |
| Dragonfly larvae associated circular virus-2 | YP_009001740.1 | Type A | Env |
| Dragonfly larvae associated circular virus-4 | YP_009001744.1 | Type A | Env |
| Porcine stool-associated circular virus 4 | YP_009021874.1 | Type A | Env |
| Sewage-associated circular DNA virus-19 | YP_009116903.1 | Type A | Env |
| Sewage-associated circular DNA virus-23 | YP_009116911.1 | Type A | Env |
| *C. sapidus* Atlantic Blue Crab associated circular virus (I0056) | Pending | Type A | This Study |
| Fiddler Crab associated circular virus (I0086a) | Pending | Type A | This Study |
| Fiddler Crab associated circular virus (I0086b) | Pending | Type A | This Study |
| *Gammarus sp.* Amphipod associated circular virus (I0153) | Pending | Type A | This Study |
| Hermit Crab associated circular virus (I0085A4) | Pending | Type A | This Study |
| *L. variegatus* Variable Sea Urchin associated circular virus (I0021) | Pending | Type A | This Study |
| *Littorina sp.* Snail associated circular virus (I0041) | Pending | Type A | This Study |
| *P. diogenes* Giant Hermit Crab associated circular virus(I0004A) | Pending | Type A | This Study |
| *P. placomus* Coral associated circular virus (I0351) | Pending | Type A | This Study |
| *S. brevirostris* Brown Rock Shrimp associated circular virus (I0722) | Pending | Type A | This Study |
| Abaca bunchy top virus | YP_001661657.1 | Type A | Nano |
| Banana bunchy top virus | NP_604477.1 | Type A | Nano |
| Beet curly top Iran virus | YP_001715619.1 | Type A | Gemini |
| Spinach curly top Arizona virus | YP_004207923.1 | Type A | Gemini |
| Abutilon Brazil virus | YP_003622541.1 | Type A | Gemini |
| Ageratum leaf curl virus - [G52] | YP_133831.1 | Type A | Gemini |
| Bean dwarf mosaic virus | NP_047224.1 | Type A | Gemini |
| Beet curly top virus | NP_040559.1 | Type A | Gemini |
| Horseradish curly top virus | NP_066183.1 | Type A | Gemini |
| Spinach severe curly top virus | YP_003966135.1 | Type A | Gemini |
| Eragrostis curvula streak virus | YP_002875758.1 | Type A | Gemini |
| Bean yellow dwarf virus | NP_612220.1 | Type A | Gemini |
| Axonopus compressus streak virus | YP_009021762.1 | Type A | Gemini |
| Sugarcane white streak virus | YP_009026387.1 | Type A | Gemini |
| Oat dwarf virus | YP_001941161.1 | Type A | Gemini |
| Faba bean necrotic yellows virus | NP_619570.1 | Type A | Nano |
| Faba bean necrotic stunt virus | YP_003104738.1 | Type A | Nano |
| Milk vetch dwarf virus | NP_619767.1 | Type A | Nano |
| Pea necrotic yellow dwarf virus | YP_008992019.1 | Type A | Nano |
| Uncultured marine virus clone GOM00189 | JX904207.1 | Type A* | Env |
| Uncultured marine virus clone GOM00860 | JX904279.1 | Type A* | Env |
| Uncultured marine virus clone GOM02856 | JX904312.1 | Type A* | Env |
| Uncultured marine virus clone SI00003 | JX904394.1 | Type A* | Env |
| Uncultured marine virus clone SI00078 | JX904407.1 | Type A* | Env |
| Uncultured marine virus clone SI00094 | JX904412.1 | Type A* | Env |
| Uncultured marine virus clone SI04298 | JX904614.1 | Type A* | Env |
| Uncultured marine virus clone SOG01184 | JX904118.1 | Type A* | Env |
| Uncultured marine virus clone SOG04106 | JX904147.1 | Type A* | Env |
| Circo-like virus-Brazil hs1 | YP_009022028.1 | Type A* | Env |
| Circoviridae 10 LDMD-2013 | YP_009109662.1 | Type A* | Env |
| Circoviridae 11 LDMD-2013 | YP_009109665.1 | Type A* | Env |
| Circoviridae 13 LDMD-2013 | YP_009109669.1 | Type A* | Env |
| Circoviridae 14 LDMD-2013 | YP_009109672.1 | Type A* | Env |
| Circoviridae 16 LDMD-2013 | YP_009109678.1 | Type A* | Env |
| Circoviridae 18 LDMD-2013 | YP_009109684.1 | Type A* | Env |
| Circoviridae 6 LDMD-2013 | YP_009109646.1 | Type A* | Env |
| Circovirus-like genome BBC-A | YP_003084144.1 | Type A* | Env |
| Circovirus-like genome CB-A | YP_003084294.1 | Type A* | Env |
| Circovirus-like genome RW-D | YP_003084289.1 | Type A* | Env |
| Circovirus-like genome SAR-B | YP_003084139.1 | Type A* | Env |
| Po-Circo-like virus 21 | YP_009109618.1 | Type A* | Env |
| Rodent stool-associated circular genome virus strain RodSCV_V-69 | JF755403.1 | Type A* | Env |
| Rodent stool-associated circular genome virus strain RodSCV_V-72 | JF755411.1 | Type A* | Env |
| Rodent stool-associated circular genome virus strain RodSCV_V-81 | JF755412.1 | Type A* | Env |
| Rodent stool-associated circular genome virus strain RodSCV_V-84 | JF755413.1 | Type A* | Env |
| Rodent stool-associated circular genome virus strain RodSCV_V-97 | JF755414.1 | Type A* | Env |
| Beak and feather disease virus | NP_047277.1 | Type B | Circo |
| Uncultured marine virus clone SI00292 | JX904424.1 | Type B | Env |
| Avon-Heathcote Estuary associated circular virus 13 | YP_009126904.1 | Type B | Env |
| Avon-Heathcote Estuary associated circular virus 26 | YP_009126878.1 | Type B | Env |
| Avon-Heathcote Estuary associated circular virus 4 | YP_009126883.1 | Type B | Env |
| Diporeia sp. associated circular virus isolate LM3487 | AGG39812.1 | Type B | Env |
| Dragonfly circularisvirus | YP_009021242.1 | Type B | Env |
| Dragonfly larvae associated circular virus-1 | YP_009001738.1 | Type B | Env |
| Dragonfly larvae associated circular virus-10 | YP_009001755.1 | Type B | Env |
| Dragonfly larvae associated circular virus-7 | YP_009001749.1 | Type B | Env |
| Finch circovirus | YP_803551.1 | Type B | Env |
| Fur seal faeces associated circular DNA virus | YP_009021878.1 | Type B | Env |
| McMurdo Ice Shelf pond-associated circular DNA virus-1 | YP_009047127.1 | Type B | Env |
| McMurdo Ice Shelf pond-associated circular DNA virus-4 | YP_009047136.1 | Type B | Env |
| Odanata-associated circular virus-12 | AJD07480.1 | Type B | Env |
| Odanata-associated circular virus-15 | AJD07487.1 | Type B | Env |
| Odanata-associated circular virus-17 | AJD07491.1 | Type B | Env |
| Odanata-associated circular virus-20 | AJD07503.1 | Type B | Env |
| Sewage-associated circular DNA virus-21 | YP_009116907.1 | Type B | Env |
| Uncultured marine virus clone GOM00443 | JX904231.1 | Type B | Env |
| *Aiptasia sp.* Sea Anemone associated circular virus (I0007C2) | Pending | Type B | This Study |
| *Aiptasia sp.* Sea Anemone associated circular virus (I0007C3) | Pending | Type B | This Study |
| *C. ornatus* Ornate Blue Crab associated circular virus (I0054) | Pending | Type B | This Study |
| *Calanoid sp.* Copepod associated circular virus (I0298) | Pending | Type B | This Study |
| *Didemnum sp.* Sea Squirt associated circular virus (I0026A4) | Pending | Type B | This Study |
| *Didemnum sp.* Sea Squirt associated circular virus (I0026A7) | Pending | Type B | This Study |
| *F. duorarum* Pink Shrimp associated circular virus (I0066) | Pending | Type B | This Study |
| *F. duorarum* Pink Shrimp associated circular virus (I0069) | Pending | Type B | This Study |
| Hermit Crab associated circular virus (I0085A5) | Pending | Type B | This Study |
| Marine Snail associated circular virus (I0084) | Pending | Type B | This Study |
| *Mytilus sp.* Clam associated circular virus (I0169) | Pending | Type B | This Study |
| *P. intermedius* Brackish Grass Shrimp associated circular virus (I0059) | Pending | Type B | This Study |
| *P. kadiakensis* Mississippi Grass Shrimp associated circular virus (I0099) | Pending | Type B | This Study |
| *Palaemonete sp.* Common Grass Shrimp associated circular virus (I0006H) | Pending | Type B | This Study |
| Chilli leaf curl virus | NP_803554.1 | Type B | Gemini |
| Cotton leaf curl Kokhran virus | NP_795347.1 | Type B | Gemini |
| Tomato pseudo-curly top virus | NP_620732.1 | Type B | Gemini |
| Turnip curly top virus | YP_003778175.1 | Type B | Gemini |
| Uncultured marine virus clone SI03705 | JX904561.1 | Type B* | Env |
| bovine stool/BK/KOR/2011 isolate CP11-49-3 | JN634851.1 | Type B* | Env |
| Circoviridae 3 LDMD-2013 | YP_009109637.1 | Type B* | Env |
| Circoviridae 5 LDMD-2013 | YP_009109641.1 | Type B* | Env |
| Circovirus-like genome CB-B | YP_003084295.1 | Type B* | Env |
